# Ten Years of *BrainAGE* as a Neuroimaging Biomarker of Brain Aging: What Insights Have We Gained?

**DOI:** 10.3389/fneur.2019.00789

**Published:** 2019-08-14

**Authors:** Katja Franke, Christian Gaser

**Affiliations:** ^1^Structural Brain Mapping Group, Department of Neurology, University Hospital Jena, Jena, Germany; ^2^Department of Psychiatry, University Hospital Jena, Jena, Germany

**Keywords:** brain age estimation, biomarker, intervention, metabolic health, MRI, neurodegeneration, neurodevelopment, psychiatric disorders

## Abstract

With the aging population, prevalence of neurodegenerative diseases is increasing, thus placing a growing burden on individuals and the whole society. However, individual rates of aging are shaped by a great variety of and the interactions between environmental, genetic, and epigenetic factors. Establishing biomarkers of the neuroanatomical aging processes exemplifies a new trend in neuroscience in order to provide risk-assessments and predictions for age-associated neurodegenerative and neuropsychiatric diseases at a single-subject level. The “*Brain Age Gap Estimation (BrainAGE)”* method constitutes the first and actually most widely applied concept for predicting and evaluating individual brain age based on structural MRI. This review summarizes all studies published within the last 10 years that have established and utilized the *BrainAGE* method to evaluate the effects of interaction of genes, environment, life burden, diseases, or life time on individual neuroanatomical aging. In future, *BrainAGE* and other brain age prediction approaches based on structural or functional markers may improve the assessment of individual risks for neurological, neuropsychiatric and neurodegenerative diseases as well as aid in developing personalized neuroprotective treatments and interventions.

## Introduction

With population growth and prolonged lifespan, the numbers of individuals with a range of (non-fatal, but) disabling disorders, including neurodegenerative diseases such as cognitive decline and dementia, are rising (1). Understanding the links between brain aging processes and neurodegenerative disease mechanisms is an urgent priority for health systems in order to establish effective strategies to deal with the rising burden. Aging is broadly defined as a time-dependent functional decline, driven by a progressive accumulation of cellular damage throughout life (2) and changes in intercellular communication (3–6). Aging is also a vastly complex process, which is individually modified by manifold genetic and environmental influences (5).

The assessment of the individual's “biological age” was recently promoted, resulting from the interaction of genes, environment, lifestyle, health, and life time, in order (i) to identify subject-specific health characteristics as well as subject-specific risk patterns for various age-related diseases based on pre-established reference curves for healthy aging, and (ii) to develop and monitor (clinical) interventions that are personally tailored based on “biological age” instead of chronological age (7). Several cell-, tissue- or function-based biomarkers that measure differences in the individual aging processes have been developed recently in order to identify and predict individual risks for age-associated diseases and mortality [for recent reviews see (8, 9)], as well as to improve intervention and treatment strategies (2, 5), including DNA methylation status, measuring the accumulation of genetic damage (7, 10, 11), telomere length, assessing telomere attrition (12–16), physical fitness, and allostatic load as a measure for physical, physiological, and metabolic health etc. (17, 18).

Structural brain maturation/aging in humans is characterized by region-specific, non-linear patterns of very well-coordinated and sequenced occurrences of progressive and regressive processes (19) / atrophy (20, 21), respectively, demonstrating robust patterns of alterations (22, 23), where some brain regions are showing greater alterations than others. With the advent of non-invasive methods of *in vivo* brain imaging, especially magnetic resonance imaging (MRI), and the availability of sophisticated computational methods for processing and analyzing MRI data, cross-sectional as well as longitudinal neuroimaging studies on brain structure and function are increasingly contributing to a more profound understanding of healthy as well as diseased structural brain maturation and aging for recent reviews see (8, 9).

As research increasingly focuses on the interplay between aging and disease, a growing body of research utilizes neuroimaging to develop a biomarker of individual brain health, so-called “brain age.” Lately, data-driven learning methods, including cross-validation, pattern classification, and regression-based predictive analyses, exemplify a new trend in biomedical and neuroscientific research, allowing measurements and predictions even at the single subject level (24). To determine the individual trajectory of brain maturation and aging as well as the risks for cognitive dysfunction and age-associated brain diseases, a number of structural and functional brain-based prediction methods for age or cognitive state enjoy increasing popularity in (cognitive) neuroscience, providing personalized biomarkers of brain structure, and function by identifying deviations from pre-established reference curves or automatically discriminating patients with brain disorders from healthy controls (25–30). Most of these studies are using state-of-the-art machine learning techniques to make predictions at the single-subject level. Especially pattern recognition and regression-based computational modeling methods aim to predict the values of continuous variables, like structural brain age, cognitive states, or neuropsychological characteristics (27). These new brain-based biomarkers offer a powerful strategy for using neuroscience in clinical practice and have a wide range of implementations, such as providing reference curves for healthy brain maturation/aging, predicting personalized brain maturation/aging trajectories, discovering protective, and harmful environmental influences on brain health, disentangling age-related from disease-specific changes in individual brain structure, aiding in the risk-assessment, and early detection of certain neurodegenerative diseases, tracking individual disease progression, as well as determining the individual relationship of structural brain aging to cognitive performance and neuropsychiatric symptoms (8).

The “*brain age gap estimation (BrainAGE)”* method, which utilizes structural MRI data to directly quantify acceleration or deceleration of individual brain aging, was the first brain aging estimation approach that (1) established reference curves for healthy brain maturation during childhood into young adulthood and for healthy brain aging during adulthood into senescence, (2) examined deviations of individual brain aging from the established reference curve of healthy brain aging in neurodegenerative diseases, (3) analyzed longitudinal changes of individual brain aging in several samples, (4) used deviations of individual brain age predictions from the established reference curve of healthy brain aging to predict worsening of cognitive functions and conversion to Alzheimer's disease (AD), (5) studied the effects of a number of several health- and lifestyle-related factors on individual brain aging, (6) monitored the effects of protective interventions on individual brain aging, and (7) was adapted to be also applied in experimental studies with rodents and nun-human primates. This review firstly describes the generation of the *BrainAGE* model and secondly recapitulates and integrates all studies predicting individual brain age with the innovative *BrainAGE* method in healthy and diseased populations. Wherever possible, studies applying other brain age prediction approaches to examine the very issue are additionally included in this review. A short summary of all *BrainAGE* studies summarized here can be found in Table 1.

**Table 1:** Studies utilizing the *BrainAGE* model for analyzing individual brain aging.

**Study focus**	**Study sample**					**Main study results^$^**
	**Groups**	**No. of subjects [female]**	**Age mean ± SD [range] in years**	**MRI [no.]**	**Mean *BrainAGE* (SD) in years**	
**EVALUATION OF** ***BRAINAGE*** **PREDICTION PERFORMANCE IN REFERENCE SAMPLES**						
Performance of the *BrainAGE* model for brain maturation during childhood & adolescence^a^	CTR	394 [47%]	10.7 ± 3.8 [5 – 19]	1.5T [6]	–	Brain age estimation was highly accurate (*r =* 0.93; *p <* 0.001). The 95% confidence interval for the prediction of brain age was stable across the entire age range (±2.6 years). MAE was 1.1 years. ***BrainAGE*** **model for brain maturation during childhood and adolescence explained 87% of the individual variations in brain structures**.
Performance of the *BrainAGE* model for brain aging from early into late adulthood^b^	CTR CTR	547 [56%] 108 [37%]	48 ± 17 [19 – 86] 32 ± 10 [20 – 59]	1.5T [2], 3T [1] 1.5T [1]	– –	Brain age estimation was highly accurate (*r =* 0.92; *p <* 0.001). The 95% confidence interval for the prediction of age was stable along the age range, with no broadening at old age (cf. age = 20 ± 11.6 years, age = 80 ± 11.7 years). Correlation between MAE and the true age indicated no systematical bias in the age estimations as a function of true ages (*r =* −0.015). MAE was 4.9 years. Results did not differ between genders (MAE: 5.0 years for males, 4.9 years for females; *r =* 0.9 for both genders). ***BrainAGE*** **model for brain aging during adulthood explained 85% of the individual variations in brain structures**.
Performance of the *BrainAGE* model in baboons^C^	CTR	29 [52%]	9.5 ± 4.9 [4 – 22]	3T [1]	–	Strong correlation between estimated brain age and chronological age (*r =* 0.80; *p <* 0.0001) MAE was 2.1 years. Best fit between chronological and estimated brain age was linear (*R^2^ =* 0.64; *p <* 0.0001). **With only 29 MRI data in the baboon sample, the baboon–specific** ***BrainAGE*** **framework showed very good performance, certainly improving with additional data**
Performance of the *BrainAGE* model in rodents^d^	CTR	24 (up to 13 scans; *n* = 273)	life span: 734 ± 110 days	3T [1]	–	Brain age estimation was highly accurate (*r =* 0.95; *p <* 0.0001). MAE was 49 days, which equates to an estimation error of 6% in relation to the age range Best fit between chronological and estimated brain age was linear (*R^2^ =* 0.91; *p <* 0.0001). Analyses of individual brain aging trajectories showed increasing variance at old ages. **Rodent–specific** ***BrainAGE*** **model showed excellent performances, explaining 91% of the individual variations in brain structures**.
**RELIABILITY OF** ***BRAINAGE*** **ESTIMATIONS**						
Scan-rescan-stability of *BrainAGE* estimations (same scanner)^e^	CTR, double-scanned on same scanner	20 [60%]	23.4 (4.0) [19 – 34]	1.5T [1]	1st scan: 13.8 (6.1) 2nd scan: 12.8 (5.6)	*BrainAGE* estimations from 1st and 2nd scan were strongly correlated (*r =* 0.93^***^) and showed ICC of 0.93^***^. *BrainAGE* scores linearly adjusted for the offset at each scanning time point strongly correlated with raw scores (*r =* 0.996^***^). ***BrainAGE*** **estimations within the same subjects proved to be stable across a short delay between two scans**.
Effect of MRI field strengths on stability of *BrainAGE* estimations^e^	CTR, double-scanned on 1.5T & 3T scanners	60 [63%]	75.2 (4.8) [60 – 87]	1.5T/3T [26/26]	1.5T scan: −5.9 (7.0) 3T scan: −9.1 (6.6)	*BrainAGE* estimations from 1.5T and 3T scan were strongly correlated (*r =* 0.91^***^) and showed ICC of 0.90^***^. *BrainAGE* scores, linearly adjusted for the scanner–specific offset, did not differ between scanners^***^. ***BrainAGE*** **estimations within the same subjects proved to be stable across scanners with different field strengths**.
Short-term changes of *BrainAGE* during the menstrual cycle f	CTR (naturally cycling women)	7 [100%]	[21 – 31]	1.5T [1]	Difference to scan at menses: Ovulation: −1.3 (1.2) Midluteal: 0.0 (1.6) Next menses: 0.1 (0.6)	*BrainAGE* decreased by −1.3 years^*^ from menses to ovulation. Classification analyses of data whether acquired at menses or ovulation is much more precise when based on *BrainAGE* (accuracy: 86%/AUC: 0.88) as compared to GM (57% 0.55), WM (43%/0.51), and CSF (64%/0.55) volumes^*^. Lower *BrainAGE* were correlated to higher estradiol levels (*r =* −0.42^*^), whereas progesterone levels did not correlate with individual *BrainAGE*. **The** ***BrainAGE*** **method proved to recognize short-term effects of hormones on individual brain structure**.
***BrainAGE*** **MODEL FOR BRAIN MATURATION DURING CHILDHOOD AND ADOLESCENCE**						
Effects of being born preterm on brain maturation^a^	Born preterm, before 27 weeks of gestation Born preterm, after 29 weeks of gestation	10 15	14.3 (1.4) [12 – 16] 14.7 (1.5) [12 – 16]	1.5T (1)	−2.0 (0.7) −0.4 (1.5)	Scanned between the ages of 12–16 years, *BrainAGE* were about 1.5 years lower in subjects who were born before the end of the 27th week of gestation vs. subjects who were born after the end of the 29th week of gestation^**^. **Although the mean difference in gestational age between both groups was only 5 weeks, results show a systematically lower** ***BrainAGE*** **in adolescents who were born extremely preterm, implying delayed brain maturation**.
***BRAINAGE*** **IN MILD COGNITIVE IMPAIRMENT AND ALZHEIMER'S DISEASE**						
Premature brain aging in AD^b^	CTR AD	232 [49%] 102 [54%]	76.0 (5.1) [60 – 90] 75.8 (8.2) [55 – 88]	1.5T [26]	0 10	**For people with mild AD, the mean** ***BrainAGE*** score was 10 years, implying a systematically higher estimated than chronological age based on structural MRI data^***^. *BrainAGE* estimations differed significantly between CTR/sMCI vs. pMCI/AD at baseline^*^ and follow-up^*^. Over the follow-up period of up to 4 years, *BrainAGE* remained stable for CTR (annual changing rate: 0.12) & sMCI (0.07), but increased in the pMCI (1.05) and AD (1.51), thus suggesting additional acceleration in brain aging^*^. Higher *BrainAGE* were related to worse cognitive functioning and more severe clinical symptoms at baseline (ADAS: *r =* 0.45^***^; CDR: *r =* 0.39^***^; MMSE: *r =* −0.46^***^) and at follow up (ADAS: *r =* 0.55^***^; CDR: *r =* 0.46^***^; MMSE: *r =* −0.55^***^).
Longitudinal changes of individual brain aging in CTR, MCI, AD^e^	CTR sMCI pMCI AD	108 [43%] 36 [17%] 112 [40%] 150 [49%]	Baseline: 75.6 (5.0) follow-up: 78.9 (5.0) Baseline: 77.0 (6.1) follow-up: 80.1 (6.0) Baseline: 74.5 (7.4) follow-up: 77.2 (7.6) Baseline: 74.6 (7.6) follow-up: 76.3 (7.7)	1.5T (26)	Baseline: −0.3 follow-up: −0.1 Baseline: −0.5 follow-up: −0.4 Baseline: 6.2 follow-up: 9.0 Baseline: 6.7 follow-up: 9.0	Changes in *BrainAGE* from baseline to last follow-up scan were related to worsening of cognitive functioning and clinical symptoms (ADAS: *r =* 0.30^***^; CDR: *r =* 0.27^***^; MMSE: *r =* −0.33^***^). **Results suggest structural changes that show the pattern of accelerated brain aging in pMCI and AD, accelerating even more, at the speed of 1 additional year in** ***BrainAGE*** **estimation per follow-up year in pMCI and 1.5 additional years in AD**.
Effects of APOE–genotype on longitudinal changes in CTR, MCI, AD^g^	CTR^C^ [APOE ε4 carriers] sMCI^C^ [APOE ε4 carriers] pMCI^C^ [APOE ε4 carriers] AD^C^ [APOE ε4 carriers] CTR^NC^ [APOE ε4 non-carriers] sMCI^NC^ [APOE ε4 non-carriers] pMCI^NC^ [APOE ε4 non-carriers] AD^NC^ [APOE ε4 non-carriers]	26 14 78 101 81 22 34 49	Baseline: 75.0 (5.1) follow-up: 78.2 (5.1) Baseline: 77.3 (5.6) follow-up: 80.4 (5.4) Baseline: 74.1 (6.5) follow-up: 76.7 (6.7) Baseline: 74.1 (6.8) follow-up: 75.8 (6.9) Baseline: 75.9 (4.9) follow-up: 79.1 (5.0) Baseline: 76.8 (6.5) follow-up: 79.9 (6.5) Baseline: 75.5 (9.3) follow-up: 78.1 (9.4) Baseline: 75.7 (8.9) follow-up: 77.4 (9.1)	1.5T [26]	Baseline: −0.1 (6.8) follow-up: −0.2 (7.9) Baseline: −0.9 (6.1) follow-up: 0.0 (6.0) Baseline: 5.8 (6.4) follow-up: 8.7 (7.2) Baseline: 5.8 (7.7) follow-up: 8.3 (8.0) Baseline: −1.3 (6.4) follow-up: −1.4 (6.1) Baseline: −0.9 (6.1) follow-up: −0.6 (4.8) Baseline: 5.5 (9.7) follow-up: 7.3 (10.3) Baseline: 6.2 (9.5) follow-up: 7.7 (10.1)	*BrainAGE* estimations differed significantly between CTR/sMCI vs. pMCI/AD at baseline^*^ and up to 4 years follow-up^*^, without significant effects regarding APOE ε4 status or interaction between diagnostic group and APOE ε4 status, nor particular allelic isoforms. Annual changing rates in *BrainAGE* differed significantly between CTR/sMCI vs. pMCI/AD as well as between APOE ε4 carriers vs. ε4 non-carriers^*^, with APOE ε4 carriers showing C NC C NC C increased changing rates (NO: 0.0; NO: 0.0; sMCI: 0.2; sMCI: −0.1; pMCI: 1.1; NC C NC pMCI: 0.6; AD: 1.7; AD: 0.9). Larger *BrainAGE* were significantly related to worse cognitive functioning and more sever clinical symptoms at baseline, being stronger in APOE ε4 non-carriers vs. ε4 carriers. **Results suggest structural changes that show the pattern of accelerated brain aging in pMCI and AD, accelerating even more during follow-up in pMCI and AD, with APOE** **ε4 carriers showing faster acceleration of brain aging**.
***BRAINAGE*****–BASED PREDICTION OF CONVERSION TO ALZHEIMER'S DISEASE**						
*BrainAGE*–based prediction of conversion from MCI to AD^h^	(1) sMCI (2) pMCI_early (3) pMCI_late	62 [21%] 58 [43%] 75 [36%]	76.4 (6.2) [58 – 88] 73.9 (7.0) [55 – 86] 75.2 (7.3) [56 – 88]	1.5T [26]	0.75 8.73 5.62	Predicting future conversion to AD within 12-months follow-up based on baseline *BrainAGE* (accuracy: 81%/AUC: 0.83) was significantly more accurate than predictions based on chronological age (41%/0.59), hippocampus volumes (left: 66%/0.69; right: 61%/0.67), cognitive scores (ADAS: 66%/0.80; CDR–SB: 59%/0.71; MMSE: 57% /0.69), and CSF biomarkers (T-Tau: 60%/0.60; P-Tau: 57%/0.66; Aβ42: 57%/0.58; Aβ42/P-Tau: 69%/0.65). Predicting future conversion to AD within 36-months follow-up based on baseline *BrainAGE* (accuracy: 75%/AUC: 0.78) was significantly more accurate than predictions based on chronological age (52%/0.56), hippocampus volumes (left: 61%/0.69; right: 54%/0.67), cognitive scores (ADAS: 48%/0.75; CDR–SB: 38%/0.67; MMSE: 37%/0.67), and CSF biomarkers (T-Tau: 58%/0.61; P-Tau: 43%/0.63; Aβ42: 49%/0.56; Aβ42/P-Tau: 73%/0.62). **Prognostic certainty for prediction of conversion to AD increased from 68% pre-test probability to 90% post-test probability when using** ***BrainAGE*** **(right hippocampus: 84%; left hippocampus: 85%; ADAS: 86%; CDR-SB: 68%; MMSE: 79%)**. **Each additional year in** ***BrainAGE*** **was associated with a 10% greater risk of developing AD during 36-months follow-up**.
Effects of APOE-genotype on *BrainAGE*-based prediction of conversion from MCI to AD^g^	sMCIC [APOE ε4 carriers] pMCIC_early [APOE ε4 carriers] pMCIC_late [APOE ε4 carriers] sMCINC [APOE ε4 non-carriers] pMCINC_early [APOE ε4 non- carriers] pMCINC_late [APOE ε4 non- carriers]	26 [12%] 33 [39%] 58 [38%] 36 [28%] 24 [46%] 16 [31%]	76.5 (5.2) 72.9 (6.0) 75.0 (6.4) 76.2 (6.8) 75.3 (8.3) 76.4 (10.0)	1.5T [26]	0.0 (4.4) 9.0 (6.3) 5.7 (6.0) 1.2 (4.0) 8.0 (9.2) 5.0 (7.7)	Cox regression showed higher baseline *BrainAGE* being associated with a higher risk of converting to AD independent of APOE status, with *BrainAGE* above median of 4.5 years indicating a nearly 4 times greater risk of converting to AD as compared to *BrainAGE* below median^***^^#^. Including APOE status into Cox model, the accuracy of the prediction tended to improve. APOE ε4 carriers: predicting future conversion to AD within 12-months follow-up based on baseline *BrainAGE* (accuracy: 85%/AUC: 0.88) was significantly more accurate than predictions based on chronological age (39%) or cognitive scores (ADAS: 69%; CDR-SB: 49%; MMSE: 46%). APOE ε4 carriers: predicting future conversion to AD within 36-months follow-up based on baseline *BrainAGE* (accuracy: 75%/AUC: 0.82) was significantly more accurate than predictions based on chronological age (54%) or cognitive scores (ADAS: 43%; CDR-SB: 26%; MMSE: 23%). APOE ε4 non-carriers: predicting future conversion to AD within 12-months follow-up based on baseline *BrainAGE* (accuracy: 78%/AUC: 0.75) was significantly more accurate than predictions based on chronological age (50%) or cognitive scores (ADAS: 68%; CDR SB: 67%; MMSE: 60%). APOE ε4 non-carriers: predicting future conversion to AD within 36-months follow-up based on baseline *BrainAGE* (accuracy: 74%/AUC: 0.71) was significantly more accurate than predictions based on chronological age (47%) or cognitive scores (ADAS: 64%; CDR SB: 51%; MMSE: 47%). From diagnosis at study baseline onwards, APOE ε4 carriers showed the tendency to take to convert to AD (560 ± 280 days) as compared to APOE ε4 non-carriers (471 ± 233 days)^#^. **Prediction of conversion was most accurate using** ***BrainAGE*** **as compared to neuropsychological test scores, even when including the APOE** **ε4-status**.
**EFFECTS OF PSYCHIATRIC DISORDERS ON BRAIN AGING**						
Effects of schizophrenia and bipolar disorder on brain aging^i^	CTR SZ BD	70 [43%] 45 [36%] 22 [55%]	33.8 (9.4) [22 - 58] 33.7 (10.5) [21 – 65] 37.7 (10.7) [24 – 58]	3T [1]	−0.2 (5.6) 2.6 (6.0) −1.2 (4.6)	*BrainAGE* scores were significantly higher in SZ by about 3 years^*^, but not BD patients. **Structural brain aging in bipolar disorder is comparable to healthy brain aging**. **Structural brain aging is significantly advanced in schizophrenia**.
Brain age in early stages of bipolar disorders or schizophrenia^k^	CTR SZ (FES) CTR Unaffected, high- risk for BD BD	43 [40%] 43 [40%] 60 [60%] 48 [60%] 48 [69%]	27.0 (4.4) 27.1 (4.9) 23.4 (4.9) 20.9 (4.1) 23.1 (4.5)	3T [1] 1.5T [2]	−0.01 (4.1) 2.6 (4.1) 0.2 (5.3) −1.0 (5.0) −1.0 (5.2)	*BrainAGE* scores were significantly higher in SZ by about 3 years^**^. The proportion of participants who had a greater biological than chronological age was higher in SZ (74%) than CTR (46%)^**^. *BrainAGE* was not associated with duration of illness or duration of untreated psychosis. No differences in *BrainAGE* between the SZ diagnoses. *BrainAGE* in SZ was negatively associated with GM volume diffusely throughout the brain^***^. **Structural brain aging is significantly advanced in schizophrenia** *BrainAGE* scores were comparable between unaffected, high-risk for BD, BD, and CTR participant's^#^. *BrainAGE* scores were not associated with number of episodes or hospitalizations, as we as duration of illness. **Structural brain aging in bipolar disorder and unaffected, high-risk subjects for BD is comparable to healthy brain aging**.
Obesity, dyslipidemia and brain age in first-episode psychosis^l^	CTR FEP	114 [45%] 120 [38%]	33.8 (9.4) [18 – 35] 33.7 (10.5) [18 – 35]	3T [1]	−0.2 (5.6) 2.6 (6.0)	*BrainAGE* scores were significantly associated with FEP^**^, obesity^**^, and BMI^*^. *BrainAGE* was highest in participants with a combination of FEP and obesity (3.8 years) and lowest in normal weight CTRs (−0.3 years) ^*^. Even among only FEP participants, BMI remained significantly associated with *BrainAGE*. As compared to CTRs, *BrainAGE* scores in non-medicated FEP participants were greater than in CTRs^**^, comparable to previously medicated FEP individuals, and not associated with cumulative exposure to antipsychotics (with non-medicated FEP participants not differing from the previously medicated ones in relevant clinical variables). Medication dosage at the time of scanning was not associated with *BrainAGE* or BMI. *BrainAGE* was not associated with duration of illness, duration of untreated psychosis, another health markers. **Brain structural aging is significantly advanced in medicated as well as non- medicated patients with psychosis (FEP)**. **Obesity added to advanced structural brain aging in controls as well as psychosis**.
**EFFECTS OF INDIVIDUAL HEALTH ON BRAIN AGING**						
Effects of type 2 diabetes mellitus on brain aging^m^	CTR DM2	87 [53%] 98 [46%]	65.3 (8.5) 64.6 (8.1)	3T [1]	0.0 (6.7) 4.6 (7.2)	Brain ages in DM2 were estimated 4.6 years higher than their chronological age^***^. Diabetes duration correlated positively with *BrainAGE* scores (*r =* 0.31^*^). *BrainAGE* scores in whole sample were related to fasting blood glucose (*r =* 0.34*; *BrainAGE* 1st vs. 4th quartile: 5.5 years^*^), TNFα levels (*r =* 0.29^**^), smoking duration (*r =* 0.20^**^; *BrainAGE* 1st vs. 4th quartile: 3.4 years^**^), alcohol consumption (*r =* 0.24^***^; *BrainAGE* 1st vs. 4th quartile: 4.1 years^**^). *BrainAGE* scores in whole sample were related to verbal fluency (*r =* −0.25^**^; *BrainAGE* 1^st^ vs. 4th quartile: 5.6 years^***^). *BrainAGE* scores in whole sample were related to depression scores (*r =* 0.23^*^; *BrainAGE* 1st vs. 4th quartile: 5.4 years^**^). *BrainAGE* scores were higher in males than females^**^. **Type 2 DM is associated with structural brain changes that reflect advanced brain aging**.
Longitudinal effects of type 2 diabetes mellitus on brain aging^m^	CTR DM2	13 [61%] 12 [67%]	Baseline: 69.9 (5.5) follow-up: 73.9 (5.7) Baseline: 63.3 (6.9) follow-up: 66.8 (6.7)	3T [1]	Baseline: 0.0 follow-up: 0.0 Baseline: 5.1 follow-up: 5.9	At baseline *BrainAGE* scores in DM2 subjects were 5.1 years higher than in CTR^*^. *BrainAGE* scores in CTR did not change during 3.8 ± 1.5 years follow-up. *BrainAGE* scores in DM2 subjects after 3.8 ± 1.5 years follow-up were 5.9 years higher than in CTR^*^. ***BrainAGE*** **in DM2 is increasing by 0.2 years per follow-up year**.
Gender-specific effects of health parameters on brain aging^n^	male CTR female CTR	118 110	75.8 (5.3) [60 – 88] 76.1 (4.8) [62 – 90]	1.5T [26]	0 0	39% of variance within *BrainAGE* scores were attributed to health parameters, with BMI, uric acid, GGT, DBD contributing most^***^. *BrainAGE* scores were related to BMI (*r =* 0.35^***^; *BrainAGE* 1st vs. 4th quartile: 7.5 years^***^), uric acid (*r =* 0.25^**^; *BrainAGE* 1st vs. 4th quartile: 5.6 years^*^), GGT (*r =* 0.20^*^; *BrainAGE* 1st vs. 4th quartile: 7.5 years^**^), DBD (*r =* 0.19^*^; *BrainAGE* 1st vs. 4th quartile: 6.6 years^**^). *BrainAGE* scores in “healthy” men (values below the medians of BMI, DBD, GGT, uric acid; *n =* 9) vs. men with “risky” health markers (values above the medians of BMI, DBD, GGT, and uric acid; *n =* 14): −8.0 vs. 6.7 years^*^. **In cognitively healthy elderly men, markers of the metabolic syndrome, and impaired liver and kidney functions were associated with subtle structural changes that reflect accelerated brain aging, whereas protective effects on brain aging were observed for markers of good health**. 32% of variance within *BrainAGE* scores were attributed to health parameters, with GGT, ALT, AST, vitamin B12 contributing most^**^. *BrainAGE* scores were related to GGT (*r =* 0.25^*^; *BrainAGE* 1st vs. 4th quartile: 6.1 years^**^), ALT (*r =* 0.23^*^; *BrainAGE* 1st vs. 4th quartile: 5.1 years^*^), AST (*r =* 0.20^*^; *BrainAGE* 1st vs. 4^th^ quartile: 3.1 years), vitamin B (*r =* −0.17; *BrainAGE* 1st vs. 4th quartile: 4.8 years^*^). 12 *BrainAGE* scores in “healthy” women (values below the medians of GGT, ALT, AST, vitamin B12; *n =* 14) vs. women with “risky” health markers (values above the medians of GGT, ALT, AST, vitamin B12; *n =* 13): −1.0 vs. 3.8 years.^*^ **In cognitively fit elderly women, protective effects on brain aging were observed for markers of good health**.
**PROTECTING INTERVENTIONS FOR BRAIN AGING**						
Effects of long-term meditation practice on brain aging^o^	CTR [no meditation practice] Meditators	50 [44%] 50 [44%]	51.4 (11.8) [24 – 77] 51.4 (12.8) [24 – 77]	1.5T [1]	0 −7.53	Brains of meditators (4–46 years practice, mean = 20 years) were estimated to be 7.5 years younger at age 50 than those of CTRs^*^. For every additional year over age fifty, meditators' brains were estimated to be an additional 1 month, 22 days younger than their chronological age^*^. Female brains were estimated to be 3.4 years younger than male brains^**^. **Meditation is beneficial for brain preservation, effectively protecting against age–related atrophy with a consistently slower rate of brain aging throughout life**.
Effects of making music on brain aging^p^	CTR [non-musicians] Amateur musicians Professional musicians	38 [39%] 45 [40%] 42 [48%]	25.2 (4.8) 24.3 (3.9) 24.3 (3.9)	1.5T [1]	0.48 (6.85) −4.51 (5.60) −3.70 (6.57)	Musicians had younger brains than non-musicians^**^. Small positive correlation between years of music making and *BrainAGE* score in professional musicians (*r =* 0.32*), suggesting that with increasing number of years of music making, the age-delaying effect (in professionals) might lessen. **Making music has an protecting effect on brain aging, with a stronger effect when it is not performed as a main profession, but as a leisure or extracurricular activity**.
**EFFECTS OF PRENATAL UNDERNUTRITION ON BRAIN AGING IN HUMANS AND NON-HUMAN PRIMATES**						
Gender-specific effects of prenatal under nutrition on brain aging in humans^q^	Men born before Dutch famine Men exposed to Dutch famine in early gestation Men conceived after Dutch famine Women born before Dutch famine Women exposed to Dutch famine in early gestation Women conceived after Dutch famine	14 19 19 21 22 23	68.6 (0.4) 67.4 (0.1) 66.7 (0.4) 68.7 (0.5) 67.4 (0.2) 66.7 (0.4)	3T [1]	−1.8 (3.5) 2.5 (5.2) 0.5 (4.6) −0.1 (4.3) 0.9 (4.0) −0.1 (5.3)	In men, the variance in individual *BrainAGE* scores was best explained by birth characteristics, late–life health characteristics, chronological age, and famine exposure^*^. In women, the variance in individual *BrainAGE* scores was best explained by birth characteristics, chronological age at MRI data acquisition, and famine exposure^*^. Premature brain aging by about 4 years in male offspring who had been exposed to Dutch famine during early gestation, as compared to men born before the famine. *BrainAGE* did not differ in the female sample. Cognitive and neuropsychiatric test scores in late adulthood did not differ between the famine exposure groups. **Exposure to prenatal under nutrition is associated with premature brain aging during late adulthood**.
Gender–specific effects of prenatal undernutrition on brain aging in non– human primates^C^	CTR MNR	12 [42%] 11 [45%]	4.9 (1.1) [4–7 (equiv. to human 14–24)] 5.0 (1.1) [4–7 (equiv. to human 14–24)]	3T [1]	−0.2 (1.9) [males: 0.9 (1.5)] [females: −1.6 (1.4)] 1.0 (1.8) c[males: 0.9 (2.4)] [females: 1.2 (0.8)]	Baboon *BrainAGE* based on species-specific preprocessed GM images, were significantly increased by 2.74 years in young adult female MNR subjects as compared to young adult female CTR offspring^**^, suggesting premature brain aging in female MNR offspring as a result of developmental programming due to fetal undernutrition. In males, *BrainAGE* did not differ between MNR and CTR offspring. **The effects of moderate MNR on individual brain aging occurred in the absence of fetal growth restriction or marked maternal weight reduction at birth**.

# p < 0.10;

* p < 0.05;

** p < 0.01;

*** p < 0.001;

$ bold type = main result/conclusion of the study; –,data not given or not applicable; Aβ42, β-amyloid-plaque deposition; AD, Alzheimer's disease; ADAS, Alzheimer's Disease Assessment Scale (score range 0–85); ALT, alanin-aminotransferase; AST, aspartat- aminotransferase; AUC, area under the curve (for receiver operation characteristic (ROC) analysis); BD, bipolar disorder; BMI, bodymass index; BrainAGE score, estimated brain age – chronological age; CDR-SB, Clinical Dementia Rating “sum of boxes” (score range 0–18); CSF, cerebrospinal fluid; CTR, control subjects; DM2, type 2 diabetes mellitus; DBD, diastolic blood pressure; FEP, first episode psychosis; FES, first episode schizophrenia; GGT, γ-glutamyltransferase; GM, gray matter; ICC, intra-class correlation coefficient (two-way random single measures); MAE, mean absolute error between brain age and chronological age; MCI, mild cognitive impairment; MMSE, Mini-Mental State Examination (score range 0–30); MNR, maternal nutrient restriction during pregnancy; P-Tau, phosphorylated tau; pMCI, progressive MCI (i.e., convert from MCI to AD during follow-up); pMCI_early, early converting pMCI (i.e., diagnosis was MCI at baseline but converted to AD within the first 12 months, without reversion to MCI or CTR at any available follow-up); pMCI_late, late converting MCI (i.e., diagnosis was MCI at baseline and conversion to AD was reported after the first 12 months of follow-up, without reversion to MCI or CTR at any available follow-up); sMCI: stable MCI (i.e., diagnosis is MCI at all available time points, but at least for 36 months); SZ, schizophrenia; T-Tau, total tau, WM: white matter

a Franke et al. (31);

b Franke et al. (32);

C Franke et al. (33);

d Franke et al. (34);

e Franke and Gaser (31); ^f^Franke et al. (35);

g Löwe et al. (36);

h Gaser et al. (37);

i Nenadic et al. (38);

k Hajek et al. (39);

l Kolenic et al. (40);

m Franke et al. (41);

n Franke et al. (42);

o Luders et al. (43);

p Rogenmoser et al. (44);

q *Franke et al. (33)*.

## Generation of the *BrainAGE* Model

A growing body of research is using high-dimensional neuroimaging data, i.e., often including several hundred (multi-modal) parameters per individual, and employing supervised, linear, or non-linear pattern recognition techniques in order to depict and quantify structural brain development and aging across the lifespan. In contrast to univariate approaches, multivariate analyses of individual brain structure are able to detect and quantify subtle, but widespread deviations in region- or voxelwise brain structure within the whole brain for the individual's age.

In general, the brain age prediction model needs to be trained first in order to subsequently assess a person's individual brain age. The brain age prediction model is generated by recognizing multivariate patterns of age-typical brain structure and parameters, utilizing MRI data of a large sample of (cognitively) healthy subjects. Subsequently, the age prediction model is applied in previously unseen test subjects, i.e., estimating the subject-specific brain ages utilizing their individual MRI data. The difference between a person's estimated brain age and its chronological age finally identifies the individual deviation from the typical maturation/aging trajectory.

### Pipeline for the Generation of Brain Age Estimations

**Figure 1:**
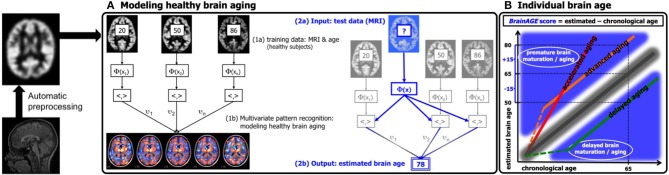
Depiction of the *BrainAGE* concept. All MRI data are automatically preprocessed via VBM. **(A)** The model of healthy brain aging is trained with the chronological age and preprocessed structural MRI data of a training sample (left; with an illustration of the most important voxel locations that were used by the age regression model). Subsequently, the individual brain ages of previously unseen test subjects are estimated, based on their MRI data. **(B)** The difference between the estimated and chronological age results in the *BrainAGE* score, with positive *BrainAGE* scores indicating advanced brain aging (orange line), increasing *BrainAGE* scores indicating accelerating brain aging (red line), and negative *BrainAGE* scores indicating delayed brain aging (green line). [Figure and legend adapted from Franke et al. (45), with permission from Hogrefe Publishing, Bern].

In general, the workflow of our innovative *BrainAGE* model includes several processing steps (Figure 1). Firstly, the raw T1-weighted image data are preprocessed with a standardized voxel-based morphometry (VBM) pipeline, resulting in comparable as well as more easily processible data to be utilized in the following analysis steps (see Preprocessing of raw MRI data). Secondly, automated data reduction of the preprocessed MRI data is performed in order to reduce computational costs, avoid method-typical over-fitting of pattern recognition, as well as to provide a robust and widely applicable age estimation model (see Data reduction). Thirdly, relevance vector regression (RVR) is performed, capturing the multidimensional maturation/aging patterns throughout the whole brain and thus modeling structural brain maturation/aging. Subsequently, individual brain ages can be estimated (see Training of the *BrainAGE* algorithm).

#### Preprocessing of Raw MRI Data

Preprocessing of the raw MRI data is done using SPM including the VBM8/CAT12 toolbox, running under MATLAB. More specifically, T1-weighted images are corrected for bias-field inhomogeneities (46, 47). Following, the images are spatially normalized. Afterwards, the images are segmented into the tree brain tissue types, i.e., gray matter (GM), white matter (WM), and cerebro-spinal fluid (CSF), within the same generative model (48). Furthermore, adaptive maximum a posteriori estimations (49) and a hidden Markov random field model (50) are applied in order to account for partial volume effects (51). Finally, image preprocessing includes affine registration.

#### Data Reduction

Preprocessed MRI data are smoothed with 4 or 8 mm full-width-at-half-maximum (FWHM) Gaussian kernels. Thereafter, data are re-sampled to 4 or 8 mm spatial resolution, resulting in 29,852 or 3,747 voxels per subject after masking out non-brain areas, respectively. Finally, principal component analysis (PCA) is applied to further reduce data dimensionality. As a great portion of the resulting voxels are still sharing much of its variances with their neighboring voxels, PCA is mathematically allowed to be performed although the numbers of data sets in the training sample is lower than the number of voxels, given the numbers of data sets in the training sample is sufficient (see Performance of the *BrainAGE* model for brain aging from early into late adulthood). The PCA model is calculated within the training data only and subsequently the resulting transformation parameters are utilized to reduce data dimensionality within the independent test samples.

#### Training of the *BrainAGE* Algorithm

The *BrainAGE* framework utilizes RVR (52, 53) with a linear kernel. Importantly, RVR does not require additional (manual) parameter optimization during the training procedure, which is advantageous over the commonly used support vector machines with regards to computational costs and robust model fitting.

In general, the age regression model is calculated within the training sample, utilizing the preprocessed structural MRI data as independent variables and the chronological ages as dependent variables, resulting in a complex model of healthy brain maturation/aging (Figure 1A, left panel). Within this specified regression task (i.e., healthy brain maturation/aging), voxel-specific weights are calculated, representing the voxel-specific importance within this regression task (for illustrations of the resulting voxel-specific weights see Figure S1 for the brain maturation model & Figure S2 for the brain aging model).

Subsequently, the brain maturation/aging model is applied to aggregate the complex, multidimensional maturation/aging pattern throughout the whole brain of a new test subject, resulting in one single value, i.e., the estimated brain age (Figure 1A, right panel).

Finally, the difference between estimated brain age and chronological age reveals the individual *brain age gap estimation* (*BrainAGE*) *score*. For *BrainAGE*, positive values are indicating advanced structural brain maturation/aging, whereas negative values are indicating delayed structural brain maturation/aging. In longitudinal studies, increasing *BrainAGE* scores are indicating accelerating brain aging over the time. Thus, the individual *BrainAGE* score is directly quantifying the amount of acceleration or deceleration of brain maturation/aging in terms of years (Figure 1B). For example, if a 70 years old individual shows a *BrainAGE* score of +5 years, the typical atrophy pattern of this individual resembles the brain structure of a 75 years old individual.

#### Cross-Validation of the *BrainAGE* Model in Reference Samples

In order to generate and validate the brain age model, most studies are employing a so-called “cross-validation” approach, i.e., the neuroimaging parameters of a large portion of the reference sample of healthy individuals are used to generate the brain age model. The generated brain age model is then applied to the smaller portion of the reference sample that was not included in the model generation step (i.e., “left-out”), in order to predict individual brain ages based on the identified neuroimaging parameters within the actual training sample. This procedure is repeated multiple times, until an individual brain age is provided for each subject in the whole reference sample.

To measure the accuracy of age estimation, Pearson's correlation coefficient (*r*), mean absolute error (MAE), and root mean squared error (RMSE) between individual estimated brain ages and chronological ages are calculated:

(1) $$\begin{array}{l} \text{MAE}={1/\text{n}}^{*}\sum_{\text{i}}|\text{B}{\text{A}}_{\text{i}}-{\text{CA}}_{\text{i}}|, \end{array}$$

(2) $$\text{RMSE}={\left[{\text{1/n}}^{*}\sum_{\text{i}}{\left({\text{BA}}_{\text{i}}-{\text{CA}}_{\text{i}}\right)}^{2}\right]}^{\text{1/2}},$$

with *n* being the number of subjects in the test sample, *BA*_*i*_ being the estimated subjects-specific brain ages, and *CA*_*i*_ being the subject-specific chronological ages. Additionally, *F* statistics of the regression model is used to analyze the fit between *BA* and *CA*.

#### Application of the Generated *BrainAGE* Model in Independent Test Samples

Additionally to the cross-validation in the reference samples, the brain age model is further validated in independent test samples of healthy and clinical subjects, in order to prove the generalizability of the pre-established brain age model across different samples and even MRI scanners, which is crucial for broad application in a clinical context, as well as to investigate the power of the brain age models as a diagnostic and prediction tool at a single-subject level, for monitoring individual changes in brain aging during treatment studies, or to explore the effects of various health characteristics, diseases, and life experiences on individual brain aging.

### Species-Specific Adaptations of the *BrainAGE* Model for Experimental Animal Studies

#### Species-Specific *BrainAGE* Model for Baboons

Within the species-specific *BrainAGE* model for baboons, we used a customized preprocessing pipeline as described in Franke et al. (33). To further reduce high-frequency noise, a spatial adaptive non-local means (SANLM) filter (54) is applied. The segmentation and spatial registration step requires a baboon-specific tissue probability map (TPM) as well as a “Diffeomorphic Anatomical Registration using Exponentiated Lie algebra” (DARTEL) template (55), which is estimated during an iterative process based on a rescaled human template. More specifically, affine transformation is initially used to scale the human SPM12 TPM and the CAT12 Dartel template map onto the brain size of baboons. Image resolution of this template is set to isotropic voxel size of 0.75 mm. For each of the performed iteration steps, the resulting tissue maps are averaged and subsequently smoothed with a 2 mm FWHM kernel to estimate an affine registration, finally resulting in a new TPM, a T1-average map, as well as a baboon-specific brain mask. To achieve averaged data, a median function is used in order to reduce distortions by outliers or failed processing. The iteration process is stopped when the actually accomplished change is below a pre-defined threshold as compared to the previous template, resulting in the final segmentation.

After Segmentation and Registration, Data are Smoothed With a 3 mm FWHM Gaussian Smoothing Kernel and re-sampled to 3 mm. Finally, PCA is Applied to Further Reduce Data Complexity (as Described in Data Reduction).

#### Species-Specific *BrainAGE* Model for Rodents

As described in Franke et al. (34), a preprocessing framework for automatically preprocessing and analyzing MRI data of rodents is providing analyses in the space of a *Paxinos* atlas (56), including several realignment and normalization steps. First, affine co-registration to the *Paxinos* template is applied utilizing normalized mutual information. In the next step, a deformation based morphometry (DBM) approach is utilized to analyze positional differences between every voxel within the actual brain data and a reference brain in order to detect structural differences over the entire brain. Thus, all measured time points of the data set of one animal are registered to the individual baseline scan. Afterwards, the deformations between all-time points and the subject-specific baseline measures are being estimated. Minimizing the morphological differences between the baseline and the follow-up brain scans, the deformation maps now encode the information about these differences. Subsequently, the Jacobian determinant of the deformations can be used to calculate local volume changes. Finally, the resulting Jacobian determinants in each voxel are filtered with a 0.4 mm FWHM Gaussian smoothing kernel.

### Technical Notes

The *BrainAGE* framework is fully automatic. All steps, including MRI preprocessing, data reduction, model training, and brain age estimation, are executed within MATLAB (www.mathworks.com). For preprocessing the T1-weighted images, SPM8 is utilized (www.fil.ion.ucl.ac.uk/spm), integrating the VBM8 toolbox (http://dbm.neuro.uni-jena.de). For the generation of brain age models in baboons and rodents our new CAT12 toolbox (http://dbm.neuro.uni-jena.de) is utilized. For PCA, the “Matlab Toolbox for Dimensionality Reduction” (https://lvdmaaten.github.io/drtoolbox/) is applied. RVR analyzes are performed utilizing the toolbox “The Spider” (http://people.kyb.tuebingen.mpg.de/spider/).

Preprocessing the human MRI data takes about 20–30 min per MRI data set on a MAC OS X, Version 10.12, 2.2 GHz Intel Core i7. The whole process of training the *BrainAGE* model and estimating brain ages takes between 1 and 5 min in total, depending on the number of features, training, and test subjects.

Baboon TPM and template generation needs about 30 min per subject and iteration, summing up in about 48 h for the whole sample of 29 control subjects. The whole process of training the baboon-specific *BrainAGE* model and estimating the individual brain ages takes about 1 min in total.

Preprocessing MRI data of rodents takes about 10–15 min per MRI data set on MAC OS X, Version 10.6.3, 2.8 GHz Intel Core 2 Duo, resulting in about 5–6 h for a sample of 24 rats with up to 13 MRI data sets per subject. Within this sample, the whole process of training the rodent-specific *BrainAGE* model and estimating the individual brain ages is performed within about 5 min.

## Evaluation of *BrainAGE* Prediction Performance in Reference Samples

### Performance of the *BrainAGE* Model for Brain Maturation During Childhood and Adolescence

**Figure 2:**
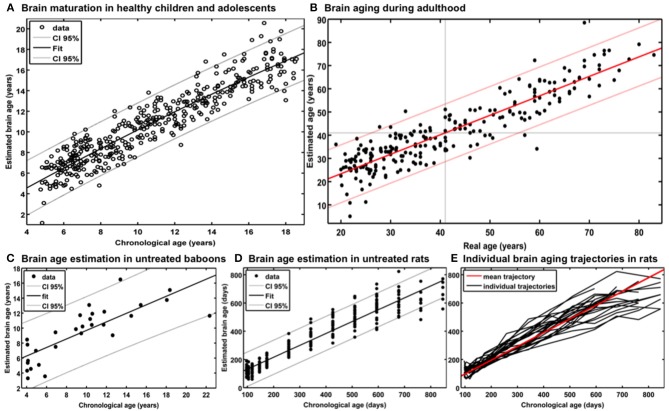
Reference curves for *BrainAGE*. **(A)** Individual structural brain age based on anatomical T1-images of 394 healthy subjects (aged 5–18 years). Chronological age is shown on the x-axis and the estimated brain age on the y-axis. The overall correlation between estimated brain age and chronological age is *r* = 0.93 (*p* < 0.001), and the overall MAE = 1.1 years. The 95% confidence interval of the quadratic fit is stable across the age range (±2.6 years). [Figure and legend reproduced from Franke et al. (45), with permission from Elsevier, Amsterdam.] **(B)** Estimated brain age and chronological age are shown for the whole test sample with the confidence interval (red lines) at a real age of 41 years of ± 11.5 years. The overall correlation between estimated brain age and chronological age is *r* = 0.92 (*p* < 0.001), and the overall MAE = 5.0 years. [Figure and legend modified from Franke et al. (32), with permission from Elsevier, Amsterdam.] **(C)** Scatterplot of estimated brain age against chronological age (in years) resulting from leave-one-out cross-validation in 29 healthy control baboons using their *in vivo* anatomical MRI scans. The overall correlation between chronological age and estimated brain age is *r* = 0.80 (*p* < 0.001), with an overall MAE of 2.1 years. [Figure and legend reproduced from Franke et al. (33), permitted under the Creative Commons Attribution License.] **(D)** (a) Chronological and estimated brain age are shown for a sample of untreated control rats, including the 95% confidence interval (gray lines). The overall correlation between chronological and estimated brain age was *r* = 0.95 (*p* < 0.0001). [Figure and legend reproduced from Franke et al. (34), with permission from IEEE.] **(E)** Longitudinal brain aging trajectories for the individual rats. [Figure and legend reproduced from Franke et al. (34), with permission from IEEE].

For generating the *BrainAGE* model during childhood and adolescence (31), GM and WM images of a cross-sectional reference sample of 394 healthy children and adolescents from the Pediatric MRI Data Repository [NIH MRI Study of Normal Brain Development; (57)] were utilized, aged 5–18 years (mean age = 10.7 years; SD = 3.9 years), with structural data acquired on six different MRI scanners (1.5T). Using leave-one-out cross-validation, the MAE between estimated brain age and chronological age was 1.1 years. Between estimated brain age and chronological age 87% of the variance were explained (*r* = 0.93; *p* < 0.001), with the 95% confidence interval being stable across the age range (± 2.6 years; Figure 2A).

Additionally, training the *BrainAGE* model with the data from only five of the six MRI scanner sites included in the study, and then applying to data from the left-out MRI scanner, estimation accuracy proved to remain stable across all scanner sites. Prediction accuracy ranged between *r* = 0.90–0.95 and MAE = 1.1–1.3 years, which proved stability of brain age estimation even across scanners (31).

A number of other studies establishing models for brain maturation including age ranges from early childhood to young adulthood have been published so far (58–63). Accuracies for brain age predictions derived from cross-validation in the reference sample ranged from *r* = 0.43–0.96 and MAEs from 1.0 to 1.9 years. The most accurate model for brain age prediction during development in healthy individuals aged 3–20 years used a number of parameters derived from different MRI modalities (i.e., T1, T2, DTI), including cortical thickness, cortical surface area, subcortical volumes, apparent diffusion coefficient, fractional anisotropy, and T2 signal intensities in predefined subcortical regions, applying a regularized multivariate non-linear regression-like approach, resulting in *r* = 0.96 and MAE = 1.0 years (59). Although each single MRI modality showed similar predictive power (r ≈ 0.9) across the full age range (i.e., 3–20 years), modality-specific contributions to the generation of the brain age model differed across neuroanatomical structures and age sub-ranges, with measures of T2 signal intensity being the strongest predictors in age 3–11 years and diffusivity measures being the strongest predictors in the ages 17–20 years (59). Additionally, modality-specific subsets showed worse prediction accuracies compared to the combined model (T1 subset: *r* = 0.91, MAE = 1.7 years; T2 subset: *r* = 0.91, MAE = 1.6 years; DTI subset: *r* = 0.90, MAE = 1.7 years). However, the *BrainAGE* method (31) outperformed all other brain age models using only a single MRI modality or single-modality subsets, and additionally proved sufficient generalizability across different scanners and even across studies.

### Performance of the *BrainAGE* Model for Brain Aging From Early Into Late Adulthood

In our first study introducing the *BrainAGE* model (32), two different samples were used to assess the brain age, i.e., the reference sample from the IXI database (www.brain-development.org; *n* = 550, aged 19–86 years, collected on three MRI scanners) and another independent test sample of healthy subjects (*n* = 108, aged 20–59 years, collected on a fourth scanner). The brain age of healthy subjects in both validation samples was accurately estimated, resulting in a MAE of 5 years and an overall correlation of *r* = 0.92, with the 95% confidence interval for the prediction of age being stable across the age range (Figure 2B). The *BrainAGE* model showed no systematical bias in MAE of brain age estimation as a function of chronological age (*r* = – 0.01). Furthermore, brain age estimation did not differ between genders (*r* = 0.92 for both genders; MAE = 5.0 years for males, MAE = 4.9 years for females).

**Figure 3:**
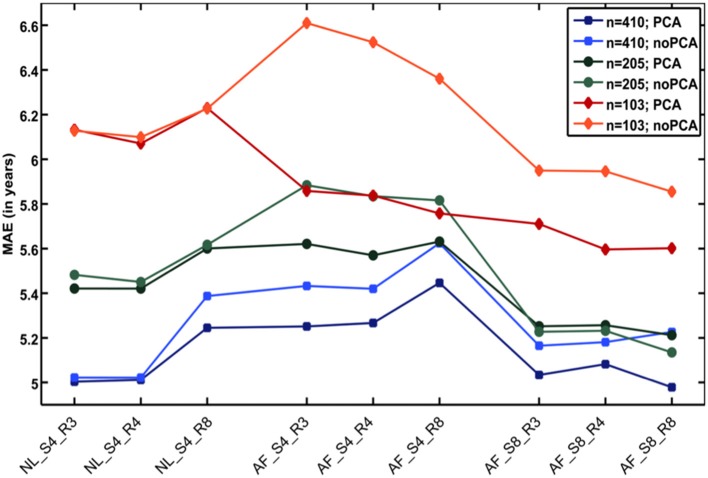
Influences of the various parameters on *BrainAGE* estimation accuracy. (1) The accuracy of age estimation essentially depends on the number of subjects used for training the age estimation model (blue lines: full training sample; green lines: ½ training sample; red lines: ¼ training sample). (2) The method for preprocessing the T1-weighted MRI images also showed a strong influence on the accuracy of age estimation. (3) Data reduction via principal component analysis (PCA) only had a moderate effect on the mean absolute error (MAE). AF, affine registration; NL, non-linear registration; R4/8, re-sampling to spatial resolution of 4/8 mm; S4/8, smoothing with FWHM smoothing kernel of 4/8 mm. [Figure and legend modified from Franke et al. (32), with permission from Elsevier, Amsterdam].

Additional analyses showed that the number of subjects in the reference sample has the strongest influence on brain age prediction accuracy, even though the choice of the preprocessing approach and model-training algorithm would also influence model performance as well as generalizability (32). In detail, the accuracy of brain age estimation worsened with reducing the size of the training/reference sample (full data set for training the *BrainAGE* model [*n* = 410]: MAE = 5 years; ½ data set [*n* = 205]: MAE = 5.2 years; ¼ data set [*n* = 103]: MAE = 5.6 years). The results further recommend a fairly rapid preprocessing of the T1-weighted MRI images with affine registration and a rather broad smoothing kernel. Dimensionality reduction of the data via PCA moderately improved brain age estimation accuracy and generalizability, while at the same time speeding up the computing time for generating the *BrainAGE* model as well as estimating the individual brain age values of the independent test subjects (Figure 3).

A number of other studies establishing models for brain aging have been published so far (55, 60, 64–79). Accuracies for brain age predictions derived from cross-validation in the whole reference sample of healthy subjects ranged from *r* = 0.43–0.97, MAEs from 4.3 to 13.5 years, and RMSEs from 5.1 to 21.0 years. In general, studies mathematically modeling healthy brain aging, which use a number of parameters derived from different MRI modalities, tended to provide more accurate brain age predictions. The best performing model in a sample of healthy participants aged 8–85 years was based on a number of T1- and DTI-derived parameters, utilizing linked independent component analysis (ICA), resulted in an overall prediction accuracy of *r* = 0.97 and MAE = 5.9 years (67). Another study also used a number of parameters derived from different MRI modalities (i.e., T1, T2, T2^*^, DTI), generating and testing their brain age model by utilizing multiple linear regression in a sample of healthy individuals aged 20–74 years, resulting in an overall age prediction accuracy of *r* = 0.96 (74). Additionally, this study found voxel-wise mean diffusivity to be the main predictor of the brain age model (i.e., explaining 62.4% of intra-individual variance), followed by GM volume (18.3%), R2^*^ (14.2%) and fractional anisotropy (3%). However, although DTI is a powerful tool offering unique information on tissue microstructure and neural fiber connections that cannot be obtained from standard structural MRI, parameters derived from DTI can differ significantly depending on the type of scanner, field strength, gradient strength, number of gradient orientations, preprocessing, fitting procedure, tractography algorithm etc. (80–83). Unfortunately, all studies including DTI failed to prove generalizability of the established brain age model in independent test samples and across scanners.

Another very recent study used a number of parameters derived from T1 and T2^*^, including cortical and subcortical measures as well as connectivity data, generating and testing the brain age model by utilizing linear support vector regression (SVR) (79). This approach showed very good performance during cross-validation within the reference sample (combined model: *r* = 0.93, MAE = 4.3 years), but a rather fair generalizability when validating the brain age model in an independent sample of healthy subjects, with data acquired on a different scanner (combined model: *r* = 0.86, MAE = 8.0 years). Aside from the *BrainAGE* approach, best prediction accuracies during cross-validation in the reference samples as well as during validation of the brain age model in independent test samples were achieved utilizing linear SVR (reference sample: *r* = 0.89, MAE = 4.3 years; independent test sample: MAE = 3.9 years; (76)], and Gaussian process regression [reference sample: *r* = 0.92, MAE = 6.2 years; independent test sample: *r* = 0.93, MAE = 5.8 years; (73)].

### Performance of the *BrainAGE* Model in Baboons

For establishing the baboon-specific brain aging model, only GM images were used. The baboon-specific brain age estimation model was trained and tested via leave-one-out cross-validation, utilizing one MRI scan per subject. Within each cross-validation loop, PCA was calculated separately in the training set and subsequently applied to the test data before performing RVR. The baboon-specific *BrainAGE* model showed very good accuracy (*r* = 0.80), with the linear regression model showing the best fit (*R*^2^ = 0.64; *p* < 0.0001; Figure 2C). Calculation of MAE resulted in 2.1 years, equating to an age estimation error of 11% in relation to the age ranged included (33, 34).

### Performance of the *BrainAGE* Model in Rodents

As described in Franke et al. (34), training and testing of the rodent-specific *BrainAGE* model was performed with subject-specific leave-one-out cross-validation processing, utilizing data sets of 24 rats, repeatedly scanned with up to 13 time points between 97 and 846 days after birth. In detail, to model the rodent-specific aging process, RVR was performed with the preprocessed structural MRI data of all scanning time points of 23 out of the total of 24 subjects. Subsequently, individual brain ages for each scanning time point of the left-out test subject were estimated, repeating the whole procedure for all 24 subjects. Brain age estimation was highly accurate (*r* = 0.95; *p* < 0.0001), with the linear regression model showing the best fit between chronological and estimated age (*R*^2^ = 0.91; *F* = 2622.3; *p* < 0.0001; Figure 2D). Mean MAE was 49 days, which equates to an error of 6% in relation to the age range within this study. Mean RMSE was 71 days. Additionally, longitudinal analyses of subject-specific brain aging trajectories revealed increasing variance between subjects in old age (Figure 2E).

## Reliability of *BrainAGE* Estimations in Healthy Adults

### Scan-Rescan-Stability of *BrainAGE* Estimations (Same Scanner)

To analyze stability and reliability of *BrainAGE* estimations, T1-weighted MRI data of 20 healthy subjects were utilized, applying the *BrainAGE* method to two MRI scans per subject, which were acquired on the same MRI scanner (1.5T) within a time period of max. 90 days. The results showed a strong scan-rescan-stability of *BrainAGE* estimations based on MRI data acquired on the same scanner, with mean *BrainAGE* scores between 1st and 2nd scan not differing among each other (*p* = 0.60) and the intra-class correlation coefficient (ICC; two-way random single measures) between *BrainAGE* scores calculated from the 1st and 2nd scan resulting in 0.93 [95% confidence interval [CI]: 0.83–0.97; (45)].

### Effect of Different MRI Field Strengths on *BrainAGE* Estimations

To analyze estimation stability across different scanners and field strengths, T1-weighted MRI data of 60 healthy subjects (aged 60–87 years) were utilized, applying the *BrainAGE* method to two MRI scans per subject, acquired on two different MRI scanners (1.5T & 3T) within a short period of time. The results suggest that the field strength affects *BrainAGE* estimations, which should be corrected for by shifting the *BrainAGE* scores to a zero group mean with a linear term in both data sets in order to gain interpretability of the results (Figure S3). After linearly adjusting for the scanner-specific offset, Student's *t*-test did not show any difference between the *BrainAGE* scores calculated from the 1.5T and 3T scans (*p* = 1.00). ICC between the *BrainAGE* scores calculated from the 1.5T and 3T scans resulted in 0.90 (CI: 0.84–0.94), demonstrating strong reliability and generalizability of the *BrainAGE* model, even with data from different scanners and field strengths (45).

### Sensitivity to Hormone-Related Short-Term Changes of *BrainAGE* in Women

**Figure 4:**
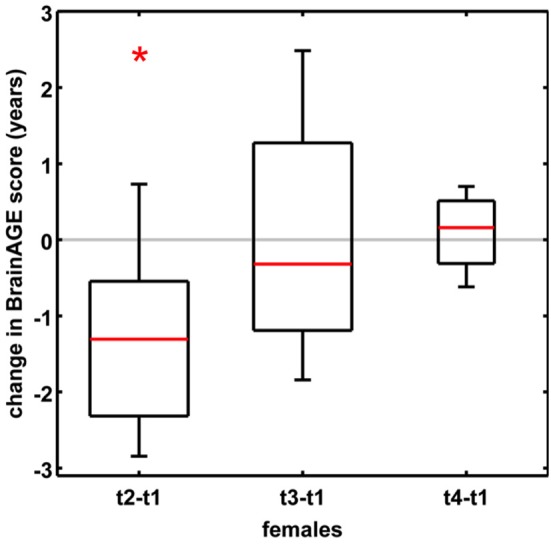
Change in *BrainAGE* scores during the menstrual cycle. *BrainAGE* scores significantly decreased by −1.3 years (SD = 1.2) at time of ovulation (i.e., t2-t1; ^*^p < 0.05). The data are displayed as boxplots, containing the values between the 25th and 75th percentiles of the samples, including the median (red lines). Lines extending above and below each box symbolize data within 1.5 times the interquartile range. The width of the boxes depends on the sample size. Note: reduced sample size at t4. [Figure and legend reproduced from Franke et al. (35), with permission from Elsevier, Amsterdam].

In order to establish the *BrainAGE* model as an innovative tool to monitor and evaluate short-term changes in individual brain aging induced by treatments and interventions, we explored its potential to recognize short-term changes in brain structure occurring during the menstrual cycle due to varying hormonal influences (35). A total of 7 young, healthy, naturally cycling women (age range 21–31 years) were scanned on a 1.5T MRI scanner (t1) during menses, (t2) at time of ovulation, (t3) in the midluteal phase, and (t4) at their next menses. During menstrual cycle *BrainAGE* scores significantly differed (*p* < 0.05), with *BrainAGE* scores decreasing by −1.3 years from menses to ovulation (SD = 1.2 years; *p* < 0.05) and after ovulation slowly increasing (Figure 4). Additionally, estradiol levels did negatively correlate with *BrainAGE* scores (*r* = −0.42, *p* < 0.05), but progesterone levels did not (*r* = 0.08, *p* = 0.71).

Another study by Luders et al. (84) explored the changes in *BrainAGE* after pregnancy. A total of 14 healthy women (aged 25–38 years) were scanned on a 3T MRI scanner within the first two after childbirth (early postpartum) as well as 4–6 weeks after childbirth (late postpartum). *BrainAGE* scores were significantly decreased by an average of −5.4 years from early to late postpartum (SD = 2.4 years; *p* < 0.001). Additional analyzes of hormone levels also showed a profound postpartum decrease in estradiol (*p* < 0.001) and progesterone (*p* < 0.001).

Taken together, these results provide strong evidence that hormonal changes during the course of the menstrual cycle have significant effects on the individual brain structure. Furthermore, the *BrainAGE* method demonstrated its potential to capture and identify subtle short-term changes in individual brain structure.

## Applications of *BrainAGE* Model for Brain Maturation During Childhood and Adolescence

### Effects of Being Born Preterm on Individual Brain Maturation

In a study with pre-term born adolescents, individual *BrainAGE* scores of subjects being born before the end of the 27th week of gestation (i.e., GA < 27; *n* = 10) were compared to those being born after the end of the 29th week of gestation (i.e., GA > 29; *n* = 15), applying the pre-established *BrainAGE* model for brain maturation during childhood and adolescence (31). At MRI scanning (1.5T), subjects were aged between 12 and 16 years. The results show significantly lower *BrainAGE* scores by 1.6 years in the group of adolescents being born GA < 27 (−1.96 ± 0.68 years) as compared to subjects being born GA > 29 (−0.40 ± 1.50 years), although the mean difference in gestation age was only 5 weeks, thus probably implying delayed structural brain maturation.

## *BrainAGE* in Mild Cognitive Impairment and Alzheimer's Disease

### Premature Brain Aging in AD

In a first proof-of-concept application, individual brain ages was studied in a group of cognitively healthy control subjects (CTR; *n* = 232) and a group of patients suffering from early Alzheimer's disease (AD; *n* = 102), applying the pre-established *BrainAGE* model for brain aging during adulthood (32). For the AD group, the mean *BrainAGE* score was +10 years (*p* < 0.001), implying systematically advanced brain aging.

**Figure 5:**
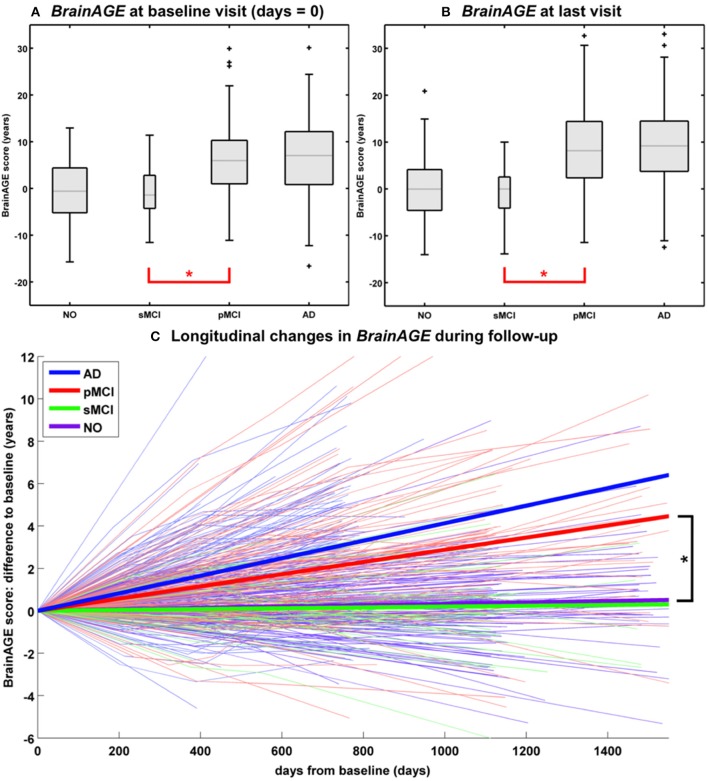
Longitudinal *BrainAGE*. Box plots of **(A)** baseline *BrainAGE* scores and **(B)** *BrainAGE* scores of last MRI scans for all diagnostic groups. *Post-hoc* t-tests showed significant differences between NO/sMCI vs. pMCI/AD (^*^p < 0.05) at both time measurements. **(C)** Longitudinal changes in *BrainAGE* scores for NO, sMCI, pMCI, and AD. Thin lines represent individual changes in *BrainAGE* over time; thick lines indicate estimated average changes for each group. *Post-hoc* t-tests showed significant differences in the longitudinal *BrainAGE* changes between NO/sMCI vs. pMCI/AD (^*^p < 0.05). [Figures and legend reproduced from Franke et al. (45), with permission from Hogrefe Publishing, Bern].

In another study that applied the pre-established *BrainAGE* model for brain aging during adulthood to data from the Alzheimer's Disease Neuroimaging Initiative (ADNI) database, baseline *BrainAGE* scores resulted in the following group means: (1) −0.3 years in CTR (i.e., being stable in the diagnosis of CTR during 36-months follow-up; *n* = 108), (2) −0.5 years in sMCI (i.e., stable MCI; being stable in the diagnosis of mild cognitive impairment (MCI) during 36-months follow-up; *n* = 36), (3) 6.2 years in pMCI (i.e., progressive MCI; changing diagnosis from MCI at baseline to AD during 36-months follow-up; *n* = 112), and (4) 6.7 years in AD (i.e., being stable in the diagnosis of AD during 36-months follow-up or until death; *n* = 150). *Post-hoc t*-tests resulted in significant *BrainAGE* differences between CTR/sMCI vs. pMCI/AD groups (*p* < 0.05), suggesting strong evidence for structural brain changes that show the pattern of advanced brain aging in the pMCI and AD groups (Figure 5A) (45).

### Longitudinal Changes of Individual Brain Aging in CTR, MCI, AD

Further analyses explored the individual brain aging trajectories in CTR, sMCI, pMCI, and AD during a follow-up period of up to 36 months (45). *BrainAGE* scores in pMCI and AD significantly increased by 1.0 additional year in brain aging per follow-up (chronological age) year in pMCI and 1.5 additional years in brain aging per follow-up (chronological age) year in AD, suggesting acceleration of individual brain aging during the course of disease (Figure 5C). With pMCI and AD subjects already showing advanced *BrainAGE* scores of about 6 to 7 years at baseline assessment and mean follow-up durations of 2.6 years for pMCI and 1.7 years for AD, mean *BrainAGE* scores at last follow-up MRI scan accumulated to about 9 years at the last MRI scan in both diagnostic groups (Figure 5B). In contrast, mean *BrainAGE* scores in CTR and sMCI subjects did not change during follow-up, thus suggesting no deviations from healthy brain aging in both groups.

Additionally, advanced structural brain aging was related to worse cognitive functioning and more severe clinical symptoms during the 36 months follow-up period (baseline *BrainAGE* scores: *r* = 0.39–0.46; *BrainAGE* scores at last follow-up visit: *r* = 0.46–0.55). Moreover, individual changes in *BrainAGE* scores were correlated with individual changes in cognitive test scores and clinical severity (*r* = 0.27–0.33), denoting a significant relationship between acceleration in individual brain aging and prospective worsening of cognitive functioning, being most pronounced in pMCI and AD subjects (45).

### Effects of APOE-Genotype on Longitudinal Changes in CTR, MCI, AD

**Figure 6:**
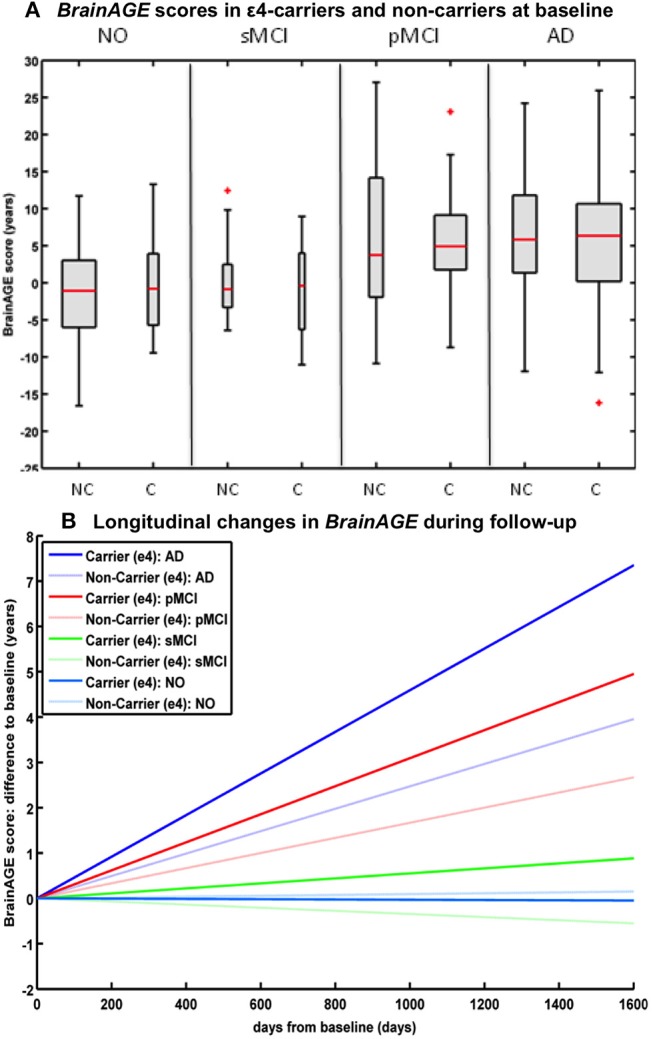
Longitudinal *BrainAGE* in APOE ε4-carriers and ε4-non-carriers. *BrainAGE* scores at **(A)** baseline for APOE ε4-carriers [C] and non-carriers [NC] in the 4 diagnostic groups NO, sMCI, pMCI, and AD. *BrainAGE* scores differed significantly between diagnostic groups (*p* < 0.001). *Post-hoc* tests showed significant differences between *BrainAGE* scores in NO as well as sMCI from *BrainAGE* scores in pMCI as well as AD (*p* < 0.05). **(B)** Estimated longitudinal changes in *BrainAGE* scores for the 4 diagnostic groups: NO (light blue), sMCI (green), pMCI (red) and AD (blue), subdivided into APOE ε4 carriers and non-carriers. *Post-hoc* t-tests resulted in significant differences for ε4 carriers and non-carriers as well as for NO/sMCI vs. pMCI/AD (*p* < 0.05). [Figures and legend reproduced from Loewe et al. (36), permitted under the Creative Commons Attribution License].

Studying the effects of Apolipoprotein E (APOE) on individual brain aging trajectories during a 36 months follow-up period, neither APOE ε4-status, nor particular allelic isoforms had a significant effect on baseline *BrainAGE* scores in the four diagnostic groups (36). However, individual brain aging accelerated significantly faster in APOE ε4-carriers as compared to APOE ε4-non-carriers in the pMCI and AD groups. More specifically, in pMCI ε4-carriers individual brain aging accelerated with the speed of 1.1 additional years per follow-up year, whereas in pMCI ε4-non-carriers individual brain aging accelerated with the speed of only about 0.6 years. Likewise, in AD ε4-carriers individual brain aging accelerated with the speed of 1.7 additional years per follow-up year, whereas in AD ε4-non-carriers individual brain aging accelerated with the speed of only about 0.9 years per follow-up year. In line with previous results, deviations from normal brain aging trajectories were not observed in healthy controls or sMCI subjects, neither in ε4-carriers nor ε4-non-carriers (Figure 6).

## *BrainAGE*-Based Prediction of Conversion to Alzheimer's Disease

### *BrainAGE*-Based Prediction of Conversion From MCI to AD

**Figure 7:**
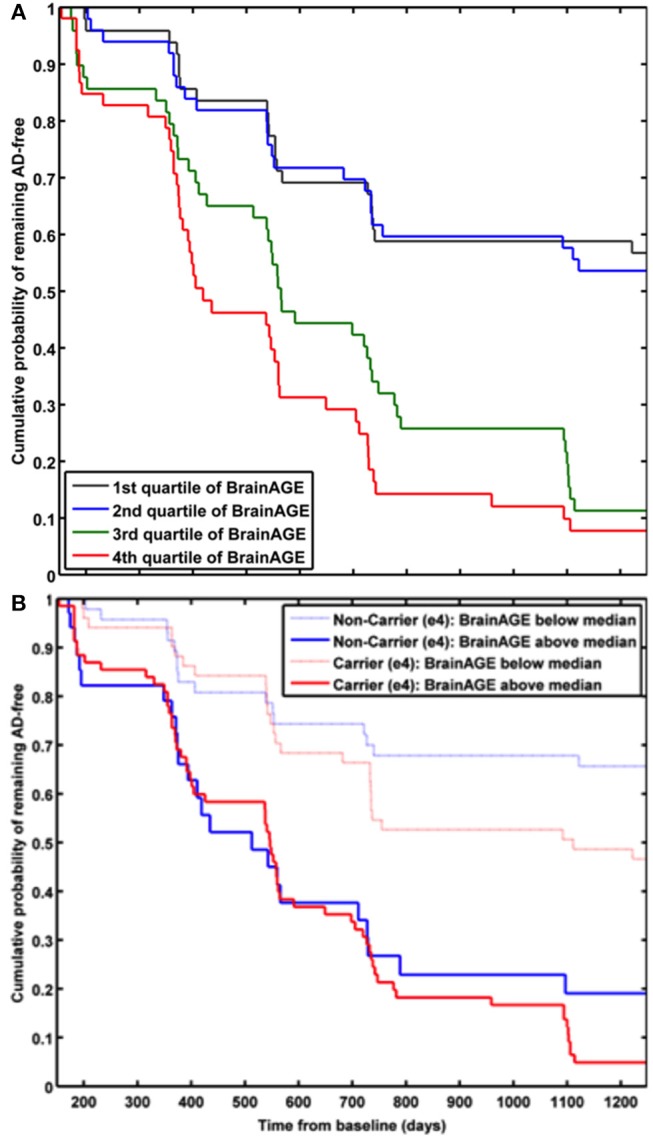
Cumulative probability for MCI patients of remaining AD-free based. **(A)** Kaplan-Meier survival curves based on Cox regression comparing cumulative AD incidence in participants with MCI at baseline by *BrainAGE* score quartiles (*p for trend* < 0.001). [Figure and legend reproduced from Gaser et al. (37), permitted under the Creative Commons Attribution License.] **(B)** Kaplan-Meier survival curves based on Cox regression comparing the cumulative incidence of AD incidence in ε4-carriers [red] and ε4-non-carriers [blue] with MCI at baseline, divided into patients with baseline *BrainAGE* scores below the median (light lines) and above the median (dark lines). Duration of follow-up is truncated at 1,250 days. [Figure and legend reproduced from Loewe et al. (36), permitted under the Creative Commons Attribution License].

In a study by Gaser et al. (37), the *BrainAGE* approach was implemented to predict future conversion to AD at a single-subject level up to 36 months in advance, based on structural MRI. The sample included 195 participants diagnosed with MCI at baseline, of whom 133 participants were diagnosed with AD during 36 months of follow-up. The *BrainAGE* scores at baseline examination differed significantly between the participants, who did not convert to AD (i.e., sMCI; 0.7 years) and those, who converted to AD within the 1st follow-up year (i.e., pMCI_fast; 8.7 years) as well as in 2nd or 3rd follow-up year (i.e., pMCI_slow; 5.6 year). A close relationship was shown between advanced brain aging, prospective worsening of cognitive functioning, and clinical disease severity. Predicting conversion from MCI to AD by using baseline *BrainAGE* scores, *post-test* probability increased to 90%. This gain in certainty based on the baseline *BrainAGE* score was 22%, being the highest as compared to baseline hippocampus volumes (right/left: 16%/17%), cognitive scores (MMSE: 11%; CDR-SB: 0%; ADAS: 18%), and even state-of-the-art CSF biomarkers (T-Tau: 4%, P-Tau: 0%, Aβ_42_: 0%, Aβ_42_/P-Tau: 8%). Predicting future conversion to AD during the 1st follow-up year based on baseline *BrainAGE* scores showed an accuracy of 81% (area under curve (AUC) in receiver-operating characteristic (ROC) analysis = 0.83), being significantly more accurate than conversion predictions based on chronological age, hippocampus volumes, cognitive scores, and CSF biomarkers (for exact numbers see Table 1). Furthermore, higher *BrainAGE* scores were related to a higher risk of developing AD, i.e., each additional year in *BrainAGE* score induced a 10% greater risk of developing AD (hazard rate: 1.1, *p* < 0.001). More specifically, as compared with participants in the lowest quartile of *BrainAGE* scores, participants in the 2nd quartile had about the same risk of developing AD (hazard ratio [HR]: 1.1; *p* = 0.68), those in the 3rd quartile had a three times greater risk (HR: 3.1; *p* < 0.001), and those in the 4th quartile had a more than four times greater risk (HR: 4.7; *p* < 0.001) of developing AD (Figure 7A). *BrainAGE* outperformed all other baseline measures.

### Effects of APOE-Genotype on *BrainAGE*-Based Prediction of Conversion From MCI to AD

A study by Loewe et al. (36) additionally explored the effects of the APOE-genotype on *BrainAGE*-based prediction of conversion from MCI to AD during the 36 months of follow-up period. Independent of APOE status, higher baseline *BrainAGE* scores were associated with a higher risk of converting to AD, with *BrainAGE* scores above median of 4.5 years resulting in a nearly 4 times greater risk of converting to AD as compared to *BrainAGE* scores below the median (HR: 3.8, *p* < 0.001). Again, the Cox regression model based on baseline *BrainAGE* scores outperformed all other models based on cognitive scores, even when including the APOE ε4-status into the models (Figure 7B). Also, predictions based on baseline *BrainAGE* scores were significantly more accurate than predictions based on chronological age or cognitive test scores (for exact numbers see Table 1), especially in APOE ε4-carriers.

## Effects of Psychiatric Disorders on Brain Aging

**Figure 8:**
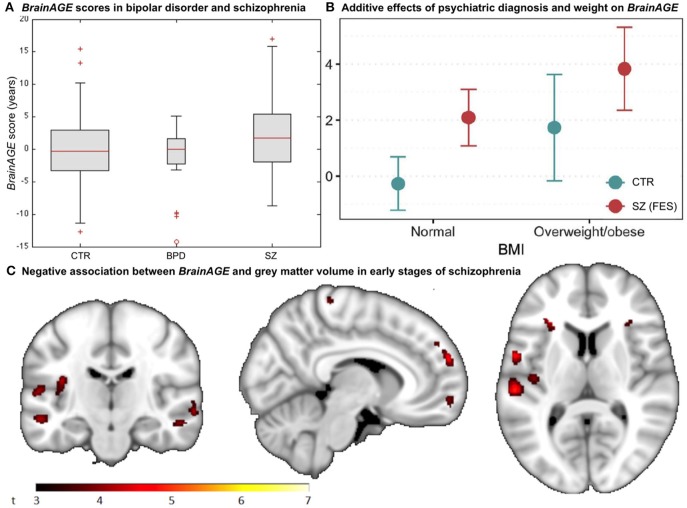
*BrainAGE* in psychiatric disorders. **(A)** Box-plot of *BrainAGE* scores in healthy controls (CTR), bipolar disorder patients (BPD), and schizophrenia patients (SZ) with significant group effect (ANOVA, *p* = 0.009), and schizophrenia patients showing higher *BrainAGE* scores than either CTR or BPD. [Figure and legend reproduced from Nenadic et al. (38), with permission from Elsevier, Amsterdam.] **(B)** Associations between *BrainAGE* scores and psychiatric diagnosis and metabolic factors. Obesity was significantly associated with *BrainAGE* scores additively to the effect of first-episode schizophrenia (FES; age adjusted mean and 95% confidence intervals). [Figure and legend reproduced from Kolenic et al. (40), with permission from Elsevier, Amsterdam.] **(C)** Negative association between *BrainAGE* and gray matter volume in participants with first episodes of schizophrenia-spectrum disorders (*P* ≤ 0.001, cluster extent = 50). [Figure and legend from Hajek et al. (39), with permission from Oxford University Press].

A recent study on the effects of psychiatric disorders on individual brain aging analyzed data from schizophrenia (SZ) patients, bipolar disorder (BD) patients (mostly with previous psychotic symptoms or episodes), as well as CTR participants, aged 21–65 years. Significantly higher *BrainAGE* scores by 2.6 years were found in SZ, but not BD patients, indicating advanced structural brain aging in SZ (Figure 8A). This study thus suggested, that there might be an additional progressive pathogenic component despite the conceptualization of SZ as a neurodevelopmental disorder (38).

Interestingly, another study by Hajek et al. (39) in young adult patients with early SZ as well as young adult patients with early BD and young adults with familial risk for BD, aged 15–35 years, resulted in comparable results. Specifically, participants with first-episode SZ showed advanced *BrainAGE* of 2.6 years as compared to their chronological age (*p* < 0.001), whereas participants at familial risk for or in the early stages of BD showed no differences between brain age and chronological age as well as compared to controls (*p* = 0.70). *Post-hoc* analyses additionally showed that *BrainAGE* was negatively associated with GM volume diffusely throughout the brain (Figure 8C). The authors concluded that the greater presence of neurostructural antecedents may differentiate SZ from BD and that *BrainAGE* could consequently aid in early differential diagnosis between BD and SZ.

A third study in first-episode SZ investigated whether comorbid obesity or dyslipidemia additionally contributes to brain alterations (40). Comparable to previous studies, young adult participants with first-episode SZ (*n* = 120; 18–35 years) showed neurostructural alterations, which resulted in their brain age exceeding their chronological age by 2.6 years (*p* < 0.001). Furthermore, the diagnosis of first-episode SZ and obesity were each additively associated with *BrainAGE* (*p* < 0.001), resulting in *BrainAGE* scores being highest in obese participants with first-episode SZ (3.8 years) and lowest in normal weight controls (−0.3 years; Figure 8B). However, neither dyslipidemia nor medical treatment was associated with *BrainAGE*. In conclusion, this study suggests obesity being an independent risk factor for diffuse brain alterations, manifesting as advanced brain aging already in the early course of SZ. Thus, targeting metabolic health and intervening at the BMI level might potentially slow brain aging in schizophrenic and psychotic patients.

## Effects of Individual Health on Brain Aging

### Effects of Type 2 Diabetes Mellitus on Brain Aging

In the study by Franke et al. (41), the *BrainAGE* method was applied to a sample of participants with type 2 diabetes mellitus (DM2) and CTR participants (mean age: 65 ± 8 years) in order to quantify the effects of DM2 on individual brain aging in cognitively healthy older adults. Participants with DM2 showed significantly increased *BrainAGE* by 4.6 years as compared to age-matched healthy CTRs (*p* < 0.001). Moreover, longer diabetes duration was correlated to higher *BrainAGE* scores (*r* = 0.31, *p* < 0.05). Additionally, *BrainAGE* scores were also positively related to fasting blood glucose (*r* = 0.34, *p* < 0.05), with a difference of 5.5 years (*p* < 0.05) between participants with the lowest vs. highest values.

### Longitudinal Effects of Type 2 Diabetes Mellitus on Brain Aging

Additionally, Franke et al. (41) further analyzed a small subsample of DM2 and CTR participants that completed a follow-up MRI scan 3.8 ± 1.5 years after their baseline assessment. GM and WM volumes did not differ between both groups or between time points. However, *BrainAGE* scores were increasing by 0.2 years per follow-up year in participants with DM2, but did not change in CTRs during follow-up. Specifically, baseline *BrainAGE* scores in DM2 patients were increased by 5.1 years as compared to CTR (*p* < 0.05), they even increased by 0.8 years during follow-up (*p* < 0.05). Thus, brain aging in DM2 did even more accelerate during follow-up.

### Individual Health and Brain Aging

**Figure 9:**
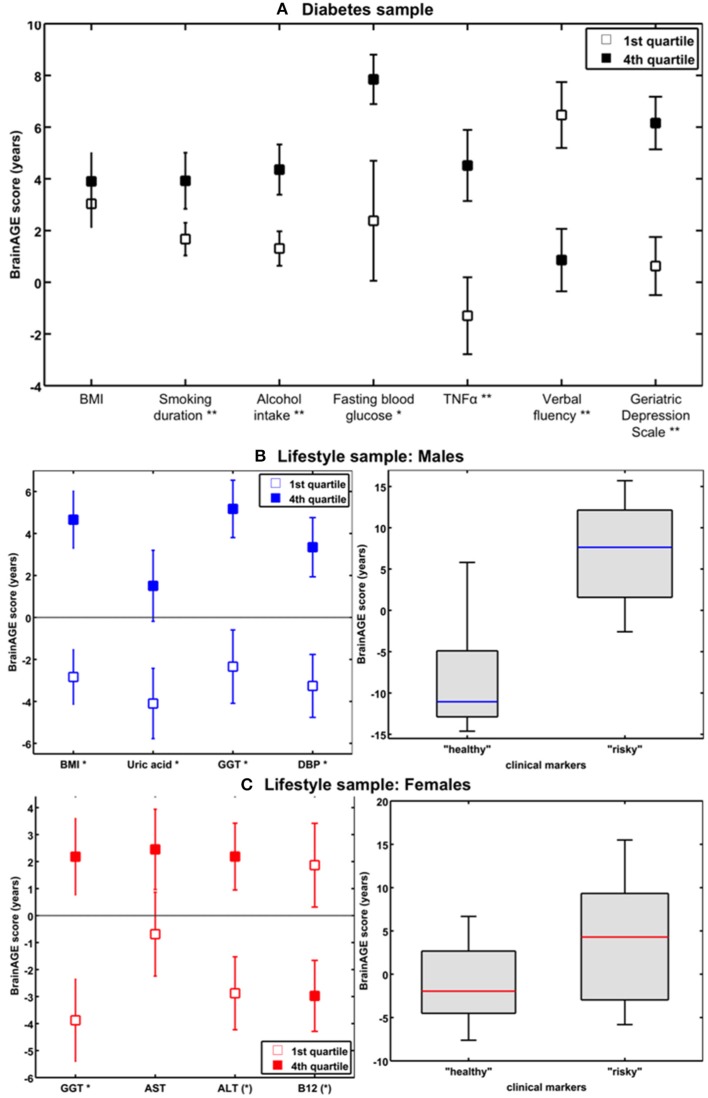
The effects of low vs. high levels in distinguished variables on *BrainAGE*. **(A)** Mean *BrainAGE* scores in participants with values in the 1st (plain squares) and 4th (filled squares) quartiles of distinguished variables from the diabetes study. [Figure and legend reproduced from Franke et al. (41), permitted under the Creative Commons Attribution License.] **(B)** Mean *BrainAGE* scores of cognitively healthy CTR men in the 1st vs. 4th quartiles of the most significant physiological and clinical chemistry parameters (left panel). *BrainAGE* scores of cognitively healthy CTR men with “healthy” markers (i.e., values below the medians of BMI, DBP, GGT, and uric acid; *n* = 9) vs. “risky” markers (i.e., values above the medians of BMI, DBP, GGT, and uric acid; n = 14; *p* < 0.05; right panel). [Figures and legend modified from Franke et al. (42), permitted under the Creative Commons Attribution License.] **(C)** Mean *BrainAGE* scores of cognitively healthy CTR women in the 1st vs. 4th quartiles of the most significant physiological and clinical chemistry parameters (left panel). *BrainAGE* scores of cognitively healthy CTR women with “healthy” markers (i.e., values below the medians of GGT, ALT, AST, and values above the median of vitamin B_12_; *n* = 14) vs. “risky” clinical markers (i.e., values above the medians of GGT, ALT, AST, and values below the median of vitamin B_12_; *n* = 13; *p* < 0.05; right panel). [Figures and legend modified from Franke et al. (42), permitted under the Creative Commons Attribution License]. ^*^p < 0.05; ^**^p < 0.01.

In addition to the effects of DM2 on individual brain aging in non-demented older adults, the study by Franke et al. (41) also explored the (additional) effects of lifestyle risk factors (i.e., smoking duration, alcohol intake), individual health marker (i.e., hypertension, TNFα), and common clinical outcomes (i.e., cognition, depression). The results revealed *BrainAGE* being also correlated to smoking duration (*r* = 0.20, *p* < 0.01), alcohol consumption (*r* = 0.24, *p* < 0.001), TNFα levels (*r* = 0.29, *p* < 0.01), verbal fluency (*r* = −0.25, *p* < 0.01), and depression (*r* = 0.23, *p* < 0.05), but not to hypertension (*p* = 0.9). Furthermore, contrasting individuals with the lowest values (i.e., 1st quartile) vs. those with the highest values in these measures (i.e., 4th quartile) resulted in *BrainAGE* differences of 3.4 years for smoking duration (*p* < 0.01), 4.1 years for alcohol intake (*p* < 0.01), 5.4 years for TNFα (*p* < 0.01), 5.6 years for verbal fluency (*p* < 0.001), and 5.4 years for depression (*p* < 0.01; Figure 9A), with all results being independent of diabetes duration, gender, and age (41).

### Gender-Specific Effects of Health Characteristics on Brain Aging

In a study by Franke et al. (42), the effects of various physiological and clinical markers of personal health on individual *BrainAGE* scores were further explored and quantified, utilizing a sample of cognitively unimpaired participants, aged 60–90 years.

In the male sample, the included health parameters explained 39% of the observed variance in *BrainAGE* (*p* < 0.001), with body mass index (BMI), uric acid, γ-glutamyl-transferase (GGT), and diastolic blood pressure (DBP) contributing most. Additional quartile analyses revealed significant differences in *BrainAGE* scores between the 1st vs. 4th quartile groups (Figure 9B, left panel), resulting in 7.5 years for BMI (*p* < 0.001), 6.6 years for DBP (*p* < 0.01), 7.5 years for GGT (*p* < 0.01), and 5.6 years for uric acid (*p* < 0.05). When combining these four health markers, the effects on individual *BrainAGE* even were compounded. In detail, comparing individual brain ages of male subjects with values below the medians vs. those with values above the medians of BMI, DBP, GGT, and uric acid resulted in *BrainAGE* scores of −8.0 vs. 6.7 years (*p* < 0.05; Figure 9B, right panel), thus suggesting a strong relationship between individual health and neurostructural aging in men.

In the female sample, the included health parameters explained 32% of the observed variance in *BrainAGE* (*p* < 0.01), with GGT, aspartat-amino-transferase (AST), alanin-amino-transferase (ALT), and vitamin B_12_ contributing most. In addition, 1st vs. 4th quartile analyses resulted in differences in *BrainAGE* (Figure 9C, left panel) of 6.6 years for GGT (*p* < 0.01), 3.1 years for AST (*p* < 0.10), 5.1 years for ALT (*p* < 0.05), and 4.8 years for vitamin B_12_ (*p* < 0.05). Again, when combining these four health markers, the effects on individual *BrainAGE* were compounded, resulting in mean *BrainAGE* scores of −1.0 vs. 3.8 years (*p* < 0.05; Figure 9C, right panel), thus suggesting a mediocre relationship between individual health and neurostructural aging in women.

## Protecting Interventions for Brain Aging

### Effects of Long-Term Meditation Practice on Brain Aging

**Figure 10:**
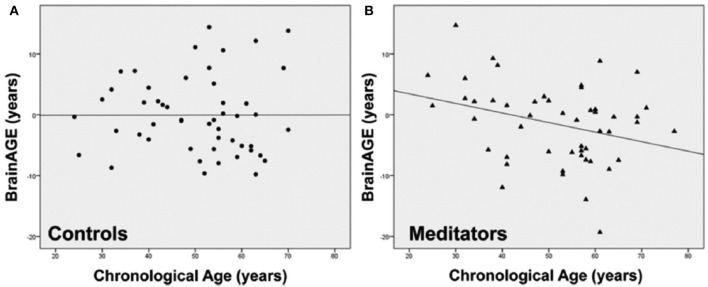
Group-specific links between age-related measures. Scatterplots and regression lines were generated separately for **(A)** controls (circles) and **(B)** meditation practitioners (triangles). The x-axes display the chronological age; the y-axes display the *BrainAGE* index (negative values indicate that participants' brains were estimated as younger than their chronological age, positive values indicate that participants' brains were estimated as older). [Figures and legend reproduced from Luders et al. (43), with permission from Elsevier].

Exploring the effects of long-term meditation practice, the study by Luders et al. (43) included 50 meditation practitioners with 4–46 years of meditation experience (mean: 20 ± 11 years) and 50 non-meditating, age-matched CTRs. At age 50 years, *BrainAGE* in meditation practitioners was about 7.5 years lower than in CTRs (*p* < 0.05). Additionally, gender exerted a main effect, with *BrainAGE* in females being lower by 3.4 years as compared to males (*p* < 0.01). Furthermore, age-by-group interaction was significant (*p* < 0.05), with follow-up analyses revealing significant effects for *BrainAGE* in meditation practitioners. In detail, for each year in chronological age over the age of 50 years, there was a significant decrease of 1 month and 22 days in *BrainAGE* in the meditation practitioners (Figure 10).

### Effects of Making Music on Brain Aging

Another study investigated the impact of music-making on brain aging, including non-musicians, amateur musicians, and professional musicians, aged 25 ± 4 years (44). All three groups were closely matched regarding age, gender, education, and other leisure activities. The “musician status” had a significant effect on *BrainAGE* (*p* < 0.05; non-musicians: −0.5 ± 6.8 years; amateur musicians: −4.5 ± 5.6 years; professional musicians: −3.7 ± 6.6 years), suggesting a decelerating effect of making music on individual brain aging. *Post-hoc* comparisons revealed lower *BrainAGE* scores in amateur musicians (*p* < 0.05) and professional musicians (*p* = 0.07) as compared to non-musicians. While no significant correlation between years involved in musical activities and *BrainAGE* score was found in amateur musicians (*r* = −0.1, *n.s*.), a small correlation was found in professional musicians (*r* = 0.3, *p* < 0.05). Thus, making music seems to have a slowing effect on the aging of the brain, especially for amateur musicians, while professional musicians revealed a lower effect probably due to stress-related interferences.

## Gender-Specific Effects of Prenatal Undernutrition on Individual Brain Aging

### Results From Studies in Humans

**Figure 11:**
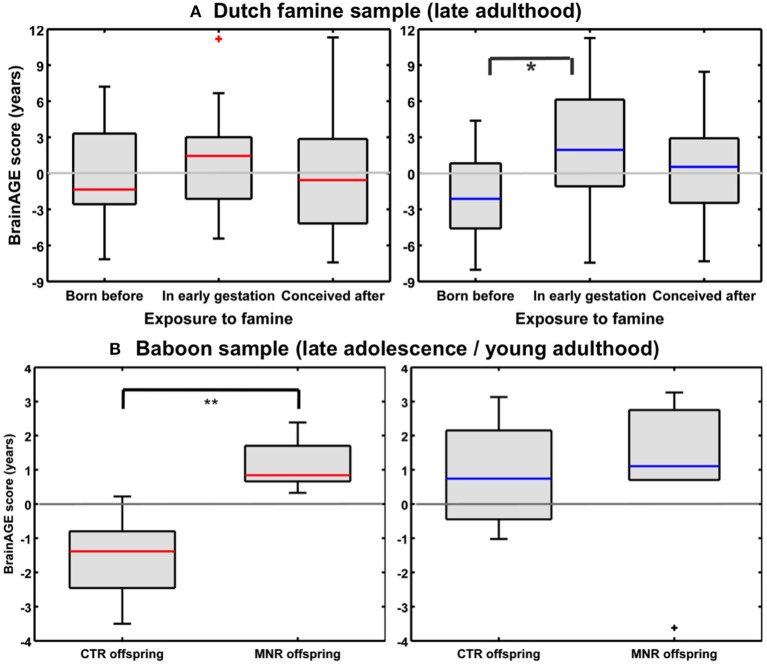
Effects of prenatal undernutrition on brain aging. **(A)** Dutch famine sample: *BrainAGE* scores in late adulthood differed significantly between the three groups only in men (blue), but not in women (red). In men, *post-hoc* tests showed significantly increased scores in those with exposure to famine in early gestation (^*^p < 0.05). [Figure and legend reproduced from Franke et al. (85), with permission from Elsevier, Amsterdam.] **(B)** Baboon sample: *BrainAGE* scores in late adolescence/young adulthood differed significantly between female (red) CTR and offspring with maternal nutrient restriction (MNR) by 2.7 years (^**^p < 0.01), but not between male (blue) CTR and MNR offspring. [Figure and legend reproduced from Franke et al. (33), permitted under the Creative Commons Attribution License].

Utilizing a subsample of the Dutch famine birth cohort, a recent study investigated the effects of fetal undernutrition during early gestation on individual brain aging in late-life (85). The participants of the MRI subsample were aged about 67 years at the time of MRI acquisition, including individuals being born before the famine in Winter 1944/45, individuals being prenatally exposed to the famine during early gestation, and individuals being conceived after the famine. In females, 28% of the observed variance *BrainAGE* at age 67 years was explained by birth characteristics, chronological age at MRI data acquisition, and famine exposure (*p* < 0.05), whereas in males, 76% the observed variance in *BrainAGE* was explained by the combination of birth characteristics, late-life health characteristics, chronological age, and famine exposure (*p* < 0.05). In the male sample, *BrainAGE* scores differed significantly between the three groups (*p* < 0.05). In the female sample, *BrainAGE* scores did not differ between the groups. *Post-hoc* tests in the male sample showed advanced brain aging by 2.5 years (*p* < 0.05) in those who had been prenatally exposed to the famine during early gestation, whereas those who had been born before the famine showed delayed brain aging by −1.8 years, resulting in a difference of about 4 years (*p* < 0.05; Figure 11A). With regard to *BrainAGE* scores there were no significant differences between males and females (85).

### Results From Studies in Non-human Primates

An experimental study of maternal nutrient restriction (MNR) in baboons also studied the effects of prenatal undernutrition on structural brain aging based on the baboon-specific *BrainAGE* model [see Species-specific *BrainAGE* model for baboons; (33)]. The experimental group included 11 subjects [5 females], with prenatal undernutrition being induced by MNR of 30% during the whole gestation. The CTR group included 12 same-aged subjects [5 females]. Subjects were aged 4–7 years [human equivalent to 14–24 years] at time of MRI data acquisition. In the female MNR offspring, baboon-specific *BrainAGE* scores were increased by 2.7 years, as compared to female CTR offspring (*p* = 0.01; Figure 11B), strongly suggesting premature brain aging resulting from prenatal undernutrition during the whole gestation. There were no differences in *BrainAGE* scores between the male MNR and CTR offspring (33).

## Summary

**Figure 12:**
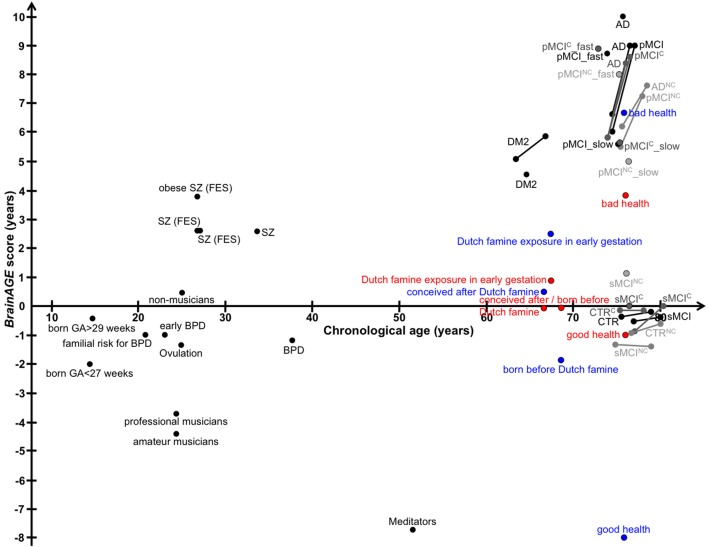
Graphical summary of *BrainAGE* results in human studies. Dots, study means; Lines, longitudinal results; Blue, males; Red, females. [AD, Alzheimer's disease; BPD, bipolar disorder; CTR, control subjects; DM2, diabetes mellitus type 2; FES, first episode of schizophrenia-spectrum disorders; GA, gestational age; MCI, mild cognitive impairment; pMCI, progressive MCI (i.e., convert from MCI to AD during follow-up); pMCI_fast, diagnosis was MCI at baseline, conversion to AD within the first 12 months (without reversion to MCI or CTR at any available follow-up; pMCI_slow, diagnosis was MCI at baseline, conversion to AD was reported after the first 12 months of follow-up (without reversion to MCI or CTR at any available follow-up); sMCI, stable MCI (i.e., diagnosis is MCI at all available time points, but at least for 36 months); SZ, schizophrenia].

In this review, we recapitulated studies that utilized the innovative *BrainAGE* biomarker to capture individual age-related brain structure, covering age ranges from childhood until late adulthood (Figure 12 for a graphic summary of all results in human studies). This predictive analytical method provides a personalized biomarker of brain structure that can help to elucidate und further examine the patterns and mechanisms underlying individual differences in brain structure and disease states. Because brain-age estimation is done on an individual level, the *BrainAGE* biomarker might be very well-suited for clinical use. The method is deriving individual predictions from multivariate patterns and interactions between voxels across the whole brain. In contrast to other structural measures, such as regional or global volumes, cortical thickness, or fractional anisotropy, *BrainAGE* scores are preserving the complex patterns of subtle variations in brain structure and their regional interactions. Additionally, reducing the complex multivariate structural information from the whole brain into a single metric resolves the problem of multiple comparisons and enables a better detection of effects (7, 24).

According to the American Federation of Aging Research (86), markers of aging should possess certain characteristics: They should be able to determine biological aging, predict the rate of aging, monitor the fundamental processes underlying aging, and be measured accurately, efficiently, and repeatedly, without harming the subject. Further, the markers need to be applicable across the species for mechanistic examinations. However, reproducibility and accuracy of some widely used biomarkers of aging, like telomere length, vary widely due to differences in extraction methods, laboratory-dependent methodological details, and measurement methods (87–89). Thus, accuracy is sometimes so low that measurement errors impede detection of differences in telomere length (88). Although biomarkers of aging should preferably be closely related to the mechanistic aging process, development of markers of brain aging that are related to brain function and structure is much more advanced and provide a considerably higher degree of correlation to age and diagnostic specificity. Moreover, brain-aging markers based on structural MRI show less inter-individual variability and methodological variations of measurements across labs or study sites. The superiority of phenotype-related markers may be explained by a number of reasons: At present, it is easier to determine phenotype because the processes underlying brain aging are complex and not yet well-understood. This is all the more so for the many compensatory pathways in the biological environment by which the organism modulates or responds to the process of aging. Aside from the complexity being present at the cellular level, the organism can respond to an infinite number of biological and environmental influences with only limited changes to the phenotype. Consequently, establishing phenotype-related biomarkers for structural brain maturation and aging (e.g., *BrainAGE*) might probably be a better approach to assess and longitudinally track individual brain aging trajectories.

In general, cognitive impairment is not due to just one disease. Cognitive impairment could be caused by AD and other forms of dementia, as well as several disease conditions, e.g., traumatic brain injury, stroke, depression, or developmental disabilities. Age-related cognitive decline is a growing concern in modern societies since mental health is perceived as a major determinant limiting quality of life during aging (90). Thus, biomarkers measuring individual brain age and predicting individual trajectory of cognitive decline are highly desirable. Approaches to determine brain age based on structural neuroimaging data are designed to indicate deviations in age-related changes in brain structure by establishing reliable reference curves for healthy brain aging and providing individual brain age measures, while accounting for the multidimensional atrophy patterns in the brain. Although multiple factors affect and modify individual brain aging trajectories, normal brain aging follows coordinated and sequenced patterns of GM and WM loss as well as CSF expansion (21, 91, 92). Several studies applying the MRI-based models for structural brain aging, have already demonstrated profound relationships between premature brain aging and AD disease severity and prospective decline of cognitive functions (45), MCI and AD (93), conversion to AD (37), SZ (76, 94), traumatic brain injury (73), HIV (95), chronic pain (96), DM2 (41), and elderly people suffering from undernutrition during gestation (85), as well as being indicative of poorer physical and mental fitness, higher allostatic load, as well as increased mortality (97). Furthermore, significant associations between individual brain aging and various health parameters, personal lifestyle, or drug use (42, 98), levels of education and physical activity (77), and meditation practice (43) have been shown. However, although Brown et al. (59) showed a relation between increased premature brain maturation and increased executive intelligence measures in adolescents as well as Steffener et al. (77) showing a correlation between delayed brain aging and higher education levels in adults, this issue has to be explored in more depth with well-characterized and well-tested samples with regards to cognitive reserve and IQ levels.

In conclusion, the phenotypic approach presented here has already established and validated reference curves for age-related changes in brain structure. Furthermore, it also showed great potential for easy application in multi-center studies. Thus, this predictive analytical method provides an individualized biomarker for determining the biological age of brain structure, which also relates to cognitive function. This MRI-based marker is able to predict individual aberrations in brain maturation and aging as well as the occurrence of age-related cognitive decline and age-related neurodegenerative diseases. This review has recapitulated evidence that neuroimaging data can be used to establish biomarkers for brain aging, which has already been confirmed as providing vital prognostic information. In future, combining different biomarkers of structural and functional brain age, like the assessment of age-related changes of parameter estimates based on the “theory of visual attention” (99–103), may enhance sensitivity and specificity for detecting aberrations in biological age compared to the chronological age in various neurological and psychiatric conditions and in neurodegenerative diseases. The important prognostic information included in the estimation of the structural and functional brain age may aid in developing personalized neuroprotective treatments and interventions.

## Author Contributions

All authors listed have made a substantial, direct and intellectual contribution to the work, and approved it for publication.

### Conflict of Interest Statement

The authors declare that the research was conducted in the absence of any commercial or financial relationships that could be construed as a potential conflict of interest.
