# Supplementary material for: Ten Years of BrainAGE as a Neuroimaging Biomarker of Brain Aging: What Insights Have We Gained?
Source: Front Neurol. 2019 Aug 14;10:789. doi: 10.3389/fneur.2019.00789 (PMC6702897; doi:10.3389/fneur.2019.00789)
Supplement: Supplementary file 2 [file Image_2.pdf]

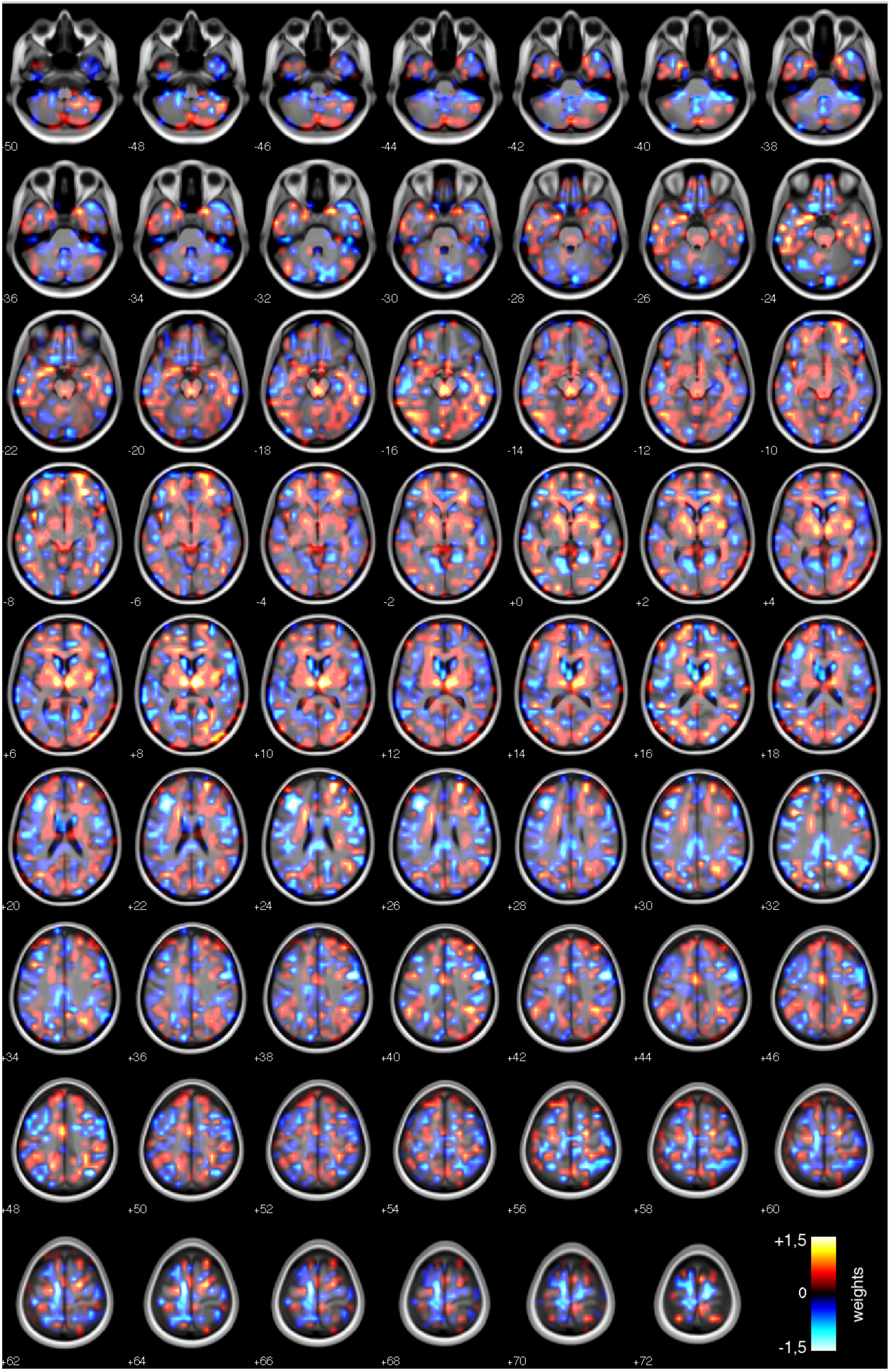

Figure S2. Exemplary GM weight map for age regression during aging in RVR. To exemplarily illustrate the most important GM features that were used by the RVR for estimating the age in adulthood based on structural MRI data, weights below the 5th and above the 95th quartile are displayed, overlaid on the normalized mean image of the sample. Color scale indicates the weight. [Figure and legend reproduced from (Franke et al., 2010), with permission from Elsevier, Amsterdam.]
