# Supplementary material for: Ten Years of BrainAGE as a Neuroimaging Biomarker of Brain Aging: What Insights Have We Gained?
Source: Front Neurol. 2019 Aug 14;10:789. doi: 10.3389/fneur.2019.00789 (PMC6702897; doi:10.3389/fneur.2019.00789)
Supplement: Supplementary file 3 [file Image_3.pdf]

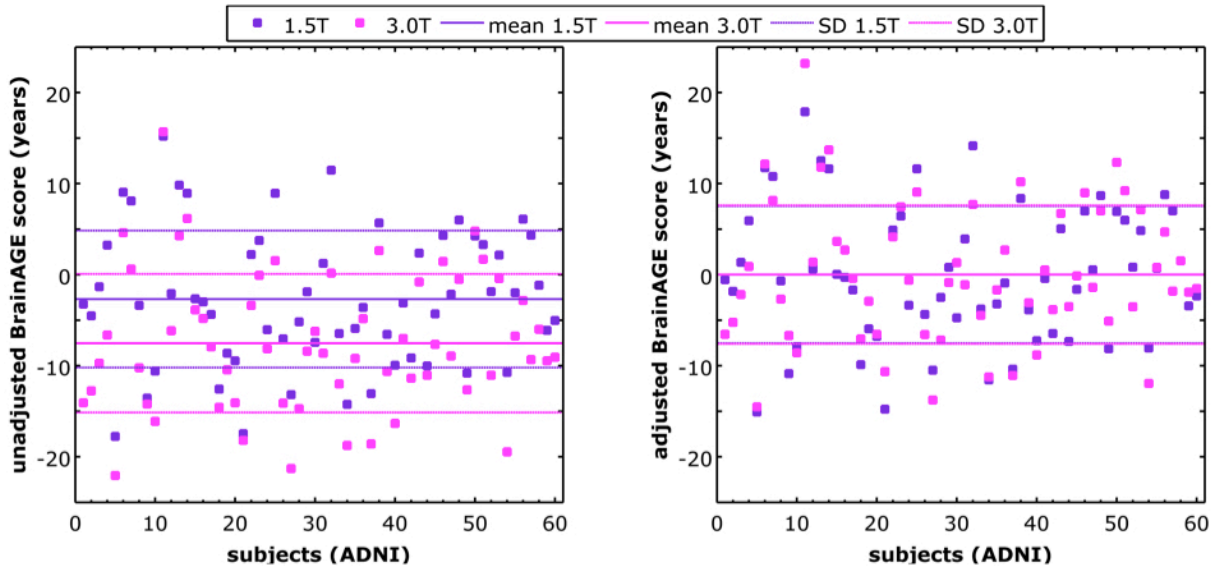

Figure S3: Scanner-specific offset-correction. Unadjusted (left panel) and scanner-specific offset-adjusted (right panel) *BrainAGE* scores for the same healthy subjects scanned on 1.5T and 3T MRI scanners within a short period of time. ICC between the *BrainAGE* scores calculated from the 1.5T and 3T scans was 0.90. [Figure and legend from Franke et al. (2012a), with permission from Hogrefe Publishing, Bern.]
